# The CODA Model: A Review and Skeptical Extension of the Constructionist Model of Emotional Episodes Induced by Music

**DOI:** 10.3389/fpsyg.2022.822264

**Published:** 2022-04-13

**Authors:** Thomas M. Lennie, Tuomas Eerola

**Affiliations:** Department of Music, Durham University, Durham, United Kingdom

**Keywords:** emotion, music, appraisal, dimensional-appraisal, constructionist, skeptical, model, goal-directed

## Abstract

This paper discusses contemporary advancements in the affective sciences (described together as skeptical theories) that can inform the music-emotion literature. Key concepts in these theories are outlined, highlighting their points of agreement and disagreement. This summary shows the importance of appraisal within the emotion process, provides a greater emphasis upon goal-directed accounts of (emotion) behavior, and a need to move away from discrete emotion “folk” concepts and toward the study of an emotional episode and its components. Consequently, three contemporary music emotion theories (BRECVEMA, Multifactorial Process Approach, and a Constructionist Account) are examined through a skeptical lens. This critique highlights the over-reliance upon categorization and a lack of acknowledgment of appraisal processes, specifically goal-directed appraisal, in examining how individual experiences of music emerge in different contexts. Based on this critique of current music-emotion models, we present our skeptically informed CODA model - Constructivistly-Organised Dimensional-Appraisal model. This model addresses skeptical limitations of existing theories, reinstates the role of goal-directed appraisal as central to what makes music relevant and meaningful to an individual in different contexts and brings together different theoretical frameworks into a single model. From the development of the CODA model, several hypotheses are proposed and applied to musical contexts. These hypotheses address theoretical issues such as acknowledging individual and contextual differences in emotional intensity and valence, as well as differentiating between induced and perceived emotions, and utilitarian and aesthetic emotions. We conclude with a sections of recommendations for future research. Altogether, this theoretical critique and proposed model points toward a positive future direction for music-emotion science. One where researchers can take forward testable predictions about what makes music relevant and meaningful to an individual.

## 1. Introduction

The discrete emotion paradigm (e.g., joy, anger, sadness) in music and emotion science has dominated theory generation and methodological practice over the last 30 years. Juslin (2019) has presented a popular interpretation of the discrete model - the BRECVEMA. Nonetheless, multiple theories representing different perspectives exist. Warrenburg's (2020) review of music-emotion theories notes remaining theoretical issues such as contextual and individual differences can be informed by direct testing of constructionist theories using emotional “granularity” of discrete terms (e.g., hot-anger, cold-anger). However, a focus solely on music-emotion theories has neglected developments in the wider affective sciences, specifically skeptical emotion theories (Moors, 2017), which emphasize the role of goal-directed appraisal in contextual and individual differences. Here, we argue that for music and emotion science to advance and address these remaining theoretical issues, the field must incorporate skeptical ideas, in turn, allowing the *relevance* and *meaning* of a stimulus to be central in music-emotion research. We assert, in line with Warrenburg and others (Céspedes-Guevara and Eerola, 2018), that a focus on context is needed for greater understanding of individual differences and the mechanisms underpinning them. However, we argue using a skeptical approach that emotion categories—“folk” terms—do not form a meaningful scientific set, undermining the study of underlying mechanisms. Instead, we seek a construction built upon the components of general cognition. Specifically, we incorporate *goal-directed appraisal* that encompasses embodied and enactive cognition within a constructionist framework. This approach allows for directly testable hypotheses, and grants a robust interpretation of relevance and meaning in musical affect.

This paper will first explore recent theoretical developments in emotion science. We will review two contemporary skeptical theories of emotion: *Psychological Constructionism* (PC, Russell, 2003, 2012), and *Dimensional-Appraisal theory* (Scherer, 2009a,b; Moors, 2013, 2014). An additional skeptical model is discussed in the Supplementary Material (Moors et al., 2017). Following this, two music specific emotion models (BRECVEMA, Juslin and Västfjäll, 2008; Juslin, 2019; and Multifactorial Process Approach, Scherer and Coutinho, 2013) are explored. A recent musical adaption of constructionist theories (Céspedes-Guevara, 2021) is additionally discussed in the Supplementary Material. We discuss the effect of skeptical theories on music-emotion models offering critiques of each from a skeptical perspective. Next, we “build on what has come before,” i.e., introduce our skeptically informed *CODA* model - a *Constructivistly-Organized Dimensional-Appraisal* model. Our model re-evaluates the role of appraisal within the music-emotion process, using a goal-directed approach that incorporates embodied and enactive forms of cognition. Moreover, it draws together competing frameworks into the same model. We outline several hypotheses regarding remaining theoretical issues, such as individual and cultural differences, perceived and induced emotions, situational effects, and one-dimensional valence. These are accompanied by new methodological techniques that can be integrated into current research paradigms. Finally, we argue the greatest benefit of adopting a skeptical approach is to move the future of music-emotion science toward the study of the emotional episodes, where the relevance and meaning of music to an individual can be studied as a dynamic process, ultimately, aligning music research with the wider field of cognitive-affective science.

## 2. Contemporary Perspectives in Emotion Psychology

We will first explore a brief critique of classical theories that skeptical theories have offered as a driving force in their construction (see also Moors's, 2017 exploration of the scientific process in emotion theory construction; for a music specific critique of classical theories see Céspedes-Guevara and Eerola, 2018). We will next explore each skeptical theory individually, identifying the alternative mechanisms they offer, notably appraisal mechanisms. Finally, we consolidate their key points of agreement and disagreement and highlight their contribution to emotion psychology.

### 2.1. Critique of Classical Emotion Theory

Classical theories of emotion (Ekman et al., 1983; Smith and Lazarus, 1991) sought to explain lexical categories as the emotional phenomena through causal mechanisms. Discrete emotions are characterized as brief, physiologically intense, pleasurable or un-pleasurable events, that are directed at something. The mechanisms to explain these phenomena can exist at different levels: observable (e.g.,stimulus input/behavioral output), cognitive (e.g., mental representations of action tendencies), or brain (e.g., neural circuits). Emotions are typically cited to include cognitive, motivational, somatic, motor, and subjective components (Frijda, 2009). We note disagreements about numbers of categories (Scherer, 2000) and locations of different components of analysis (e.g., somatic = brain level).

Classical theories each suggest different mechanisms to get from stimulus to emotion. Basic emotion theories suggest that features of evolutionary relevant stimuli, trigger evolutionary hardwired affect programs (brain level). Alternatively, classical discrete-appraisal theories suggest stimulus features are processed through cognitive evaluations that form patterns for different discrete emotions (cognitive level). Appraisal theorists disagree about whether these evaluation patterns are innate or born from a predisposition (Ellsworth and Scherer, 2003) but all discrete-appraisal theories concede that for a stimulus to generate an emotion it must impact upon a person's goals. The overlap between classical theories is that emotions (observable level) are explicitly differentiated from other phenomena through “the nature of their causal mechanisms” (Moors, 2017). A key distinction between these classical theories is the flexibility that appraisal theory offers to the emotion process.

Skeptical theories argue that discrete emotion categories have not proven to be a scientifically meaningful set (i.e., there is no deep ground that links them together) because the mechanisms proposed have not provided the necessary and sufficient conditions for a scientific set to be formed. Instead, the phenomena to be explained should be moved to the *emotional episode* (Russell, 2003, 2012). The evidence to support this claim has come from large-scale meta-analyses and methodological critiques. Against basic affect-programs, large meta-analyses focusing on psycho-physiological (Cacioppo et al., 2000) and autonomic (Quigley and Barrett, 2014; Clark-Polner et al., 2016; Siegel et al., 2018) responses for emotion categories report weak evidence for the existence of emotion circuits. In addition, cross-cultural studies in indigenous societies (Crivelli et al., 2016; Russell, 2017a) show accuracy ratings well below the predicted universality thesis 70% (Haidt and Keltner, 1999). Methodological critiques further question these already weak results. Nelson and Russell (2013) critique facial expressions paradigms highlighting the confirmatory nature of forced-choice paradigms artificially inflating results. Similar critiques have been directed toward classical-appraisal theory, both toward data (Kuppens et al., 2017) and methodology (Moors and Scherer, 2013) though no large scale meta-analyses of discrete-appraisal patterns has been conducted. Most notable is the conflation that can occur between self-reported appraisal and emotions; people may instead be expressing conceptual rather than causal relationships (Parkinson, 1997).

It follows that skeptical theories instead suggest the infinite number of possible emotions and shades of emotion are more meaningfully organized into multidimensional space. This idea however, is not incompatible with classical theories of emotion (Hu et al., 2022). Skeptical theories further reject shifting the dependent variable from discrete emotions to “granulated,” “fuzzy-set,” or “families” of emotions in which these subsets of emotion terms produce similar but not identical profiles. Skeptical theories instead argue that “organizing emotional episodes into these families is not scientifically interesting because there is no deep ground, such as a dedicated mechanism, to confer a special status to these families” (Moors, 2017). For example, subdividing the anger category into *hot-anger* and *cold-anger* as Huron (2015) suggests provides no exclusive underlying mechanistic support. It is this focus upon alternative, and importantly testable mechanisms, that marks skeptical theories as distinct. Simply, boundaries between emotions “serve no scientific purpose” (Russell, 2003; p. 1279).

### 2.2. Skeptical Theories and the Key Role of Appraisal

#### 2.2.1. Psychological Construction

Psychological Construction (PC) theory (Russell, 2003, 2012) suggests that emotion behaviors are driven by the same mechanisms as non-emotional behaviors, thus not driven by a single dedicated mechanism. It does not state what these mechanisms are and instead posits that the problem of how most emotion components are caused is a question for behavior science, while subjective experience should be understood through studies into consciousness. Russell constructs his theory around three types of affect “core-affect,” “affective quality,” and “emotional meta-experience,” corresponding to three levels of observation.

**Core-affect** is seen as an ongoing neuro-physiological process in which two dimensions, “arousal” (activation values) and “valence” (hedonic values), form a single experiential composite representative of basic feeling. Changes in core-affect are described as evoking “a search for its cause and therefore facilitating attention to and accessibility of like-valenced material. Core-affect thus guides cognitive processing according to the principle of mood congruence and is involved in motivation, reward, and reinforcement” (Russell, 2012). There are findings for multiple neural mechanisms underpinning core-affect (Posner et al., 2005) such as pleasure circuits (Berridge and Kringelbach, 2013; Kringelbach and Berridge, 2015), and a “generalized arousal system” (Pfaff, 2006). This is a positive step in linking neural circuits with core-affect for a refined definition^1^. This reasserts that emotions are distributed and cannot be localized to distinct brain networks (Barrett and Satpute, 2013) and implies a more ambitious framework than that outlined by basic emotion theory.

**Affective quality** can be conceptualized as evaluations of a stimulus (e.g., liking and attitudes). Like core-affect, affective qualities are fundamental and cannot be separated at a psychological level from other artifacts in subjective stimulus representation. Affective quality is separable from, and can occur independently of, core-affect (e.g., a cold cognitive assessment of a stimulus) and forms part of general cognition (Russell, 2003).

**Emotional meta-experience** represents the conscious experience of emotion components, or a perception of a specific emotion. It is a *meta* experience in that it includes other bottom-up and top-down experiences (e.g., core-affect, appraisal, beliefs, plans). It is stated that while there is some overlap between emotional meta-experience and emotional episode, they are not completely equivalent (Russell, 2012). A similar idea is explored by LeDoux (2008) in the concept of “feeling” where feeling must be attended to and preserved in working memory to become conscious. Yet, an emotional episode does not imply an emotional categorization or the explicit use of one or more emotion components. Simply, an emotional episode is an enclosed moment of time that is subjectively acknowledged as emotional, it has a clear on-set, and is typically directed at something. An example of an emotional episode can be seen in the classic work of William James (1884) when a bear is encountered on a walk in the woods. However, where James would have attributed the emotional experience to physiological bodily sensations, in PC no single emotion component need define the episode, what Colombetti (2014, p. 57) describes as “self-organizing patterns of the entire organism.”

Although no specific mechanisms are hypothesized by Russell to facilitate this emergence into consciousness, he suggests that core-affect's evolutionary history is linked closely to the evolution of “flexible” behavior that is not driven explicitly by the stimuli. This includes goal-setting and causal knowledge of the action to attainment link. He draws upon the work of Cabanac (2010) who argues that consciousness first emerged as an awareness of pleasure and displeasure and the work of Balleine and Dickinson (1998) who argue for consciousness as a link between *basic motivational systems* and cognitive goal-relevance systems to refine this concept.

Several criticisms of Russell's theory have emerged for one-dimensional valence such as an inability to explain mixed emotions (Hunter et al., 2008; Eerola and Peltola, 2016; Maksimainen et al., 2019); the lack of predictive power in one-dimensional valence for behavior (e.g., fear and anger may produce different approach/avoidance behaviors, Frijda, 2009) and the bivalenced conception of some affective lexis (Zeelenberg and Pieters, 2006). Russell (2012) counters this through the inclusion of multiple affective qualities (evaluations of a stimulus). However, an alternative “multifaceted mirco-valences” interpretation remains possible - i.e., the contribution of multiple “qualitatively different types of valence” (Shuman et al., 2013, 5–6).

#### 2.2.2. Dimensional-Appraisal

An alternative skeptical theory, Dimensional-Appraisal (D-A) theory (Scherer, 2009a,b; Moors, 2013, 2014), defines appraisals as cognitive mechanisms that influence other components of an emotional episode through associative motivational mechanisms. The term appraisal as a psychologically defined term was used by Arnold (Arnold, 1960) however it origins in the role of emotion go back to ancient Greece (Moors, 2013). There is a history in psychology that placed appraisal solely in the realm of the cognitive (Colombetti, 2014) however, we wish to explicitly separate the modern (dimensional) interpretation of appraisal from its predecessor (discrete). Dimensional-appraisal is distinct from discrete-appraisal in that appraisals do not form appraisal patterns that equate to discrete emotions. Instead, appraisals each contribute independently, and therefore partially, to further components of emotions (e.g., action tendencies). Each appraisal can feedforward and influence other components before other appraisal values have been generated (“immediate efference”), while each further emotional component can also feedback into appraisal mechanisms (“recurrence”^2^ Scherer, 2009b). Both of these concepts allow flexibility in the emotion process where a strict linear sequence does not have to be followed. These processes together imply appraisals can be seen as ongoing parallel processes. Appraisals can operate in both a rule-based and associative manner (Moors, 2020). Rule-based processes are typically employed the first time a stimulus is encountered while associative processes occur when the same or familiar stimuli appear. An assessment of stimulus familiarity is clearly an important determinant, yet different stances on which process takes precedence in which situations are taken by different theories.

Appraisals are typically subconscious processes. Evidence for the automatic processing of *novelty, goal-relevance, intrinsic valence, goal-congruence, control*, and *agency/intentionality* (Moors, 2020) has emerged. Features of appraisals can appear in consciousness and inform subjective experience, as can features of all components of an emotional episode. Appraisal can be seen as a general cognitive mechanism, often utilized by non-emotional behavior research (Eder and Hommel, 2013). The difference between emotional and non-emotional episodes is defined as a gradual process, mediated by an appraisal value of higher goal-relevance and a subsequent action tendency with a greater control priority. D-A theory is linked closely with Russell's (2003) concept of *affective quality* where the mechanisms (appraisals) can be conceived as ongoing non-emotional processes that under certain conditions (high goal-relevance) can produce an emotional episode.

D-A suggests that emotional components are closely related through causal mechanisms. To validate this causal-mechanistic approach D-A theorists seek to test the relationship between individual appraisal criteria and other components of emotion. For example, Moors suggests higher goal relevance would cause a greater intensity of action tendencies, while goal in/congruence would produce motivational avoidance/approach tendencies (Moors and Scherer, 2013). Hypothesis generation is still ongoing however, some authors (Scherer, 2009b) suggest that different appraisal factors receive different weightings based on other appraisal factor outputs. Stimuli are continuously reappraised through this process (*recurrence*). Identifying the underlying neurological structures of appraisals is an active area of research (Brosch and Sander, 2013; Kafkas and Montaldi, 2014). Moreover, sophisticated modeling techniques that can capture the dynamic nature of emotional processes over time have also been proposed, such as non-linear dynamic system theory (Scherer, 2009b; Colombetti, 2014). Such models have been highly successful in the neurological literature (Friston et al., 2000) and have begun to be applied to the psychological literature attempting to bridge these two disciplines (Lewis, 2005), notably by incorporating appraisal. Finally, dimensional-appraisal in its contemporary form has removed itself from its disembodied origins (Arnold, 1960) and placed neatly with contemporary understandings of meaning making such as 4E cognition Colombetti (2018).

### 2.3. Key Elements of Skeptical Theories

It can be seen that there is a substantial amount of overlap between these skeptical theories (Moors, 2017). First, all theories contend that the phenomena to be explained should be moved away from discrete emotion “folk” terms, or granular interpretations of the same concept, and should instead be placed upon the components of an emotion episode. See Barrett (2017) for a more mediated view of PC where emotion terms and their conceptual organization do form sets that are born from predictive processing of emotion components—including goals. The emotion concepts do not have a physical reality in the brain but they do have a cognitive reality, perhaps equivalent to emotion schema.

Second, these theories conclude that the components of emotion and their innumerable possible subdivisions are more logically represented by multi-dimension space. The distinction between these theories lies in how closely the components are seen as causally related and whether the individual components of an emotional episode will form a meaningful scientific set of their own. PC suggests that there is almost no relationship between these components, D-A suggests there is. The parallel-competitive dual-process model discussed in the Supplementary Material remains moot on this point.

Third, the most distinctive point across skeptical theories is the unique position that “goals”^3^ hold. From this perspective it seems strange that goal-directed accounts have had such little impact upon the science of music and emotion. This neglect is historically likely to have emerged because of the emphasis appraisal (discrete and dimensional) places upon goal-relevance, something that art/aesthetic emotions—including musical emotions—was not presumed to have. This is most evident in the pervasive disembodied Kantian notion of *disinterest*, an idea that Huron (2016) describes as incompatible with current biological stances (p. 242). In the next section we will show how goals, and appraisals generally, appear highly relevant to musical emotion process, though broadly neglected by current models. We finish this paper by taking these critiques and presenting a skeptically informed model that places appraisal back into music emotion theory.

Finally, we point the reader to other applications of skeptical theories where PC and D-A have been merged in attempts to bridge gaps between emotion and cognition literature. We offer a detailed exploration of one skeptical dual-process model in the Supplementary Material (Moors et al., 2017; Moors and Fischer, 2019).

## 3. Two Mainstream Models in Music and Emotion

Before continuing into our next section, it is important to ask why we need music specific models of emotion. The emotional power of music has long been accepted as an important element of the musical experience however, philosophers as far back as Plato and Aristotle (Gabrielsson and Lindström, 2010) up to the present day Kivy (1981, 1989) have disagreed on the how and why of this “mysterious” experience. For example, the unique experience of sad music can be untypically positive (Eerola and Peltola, 2016) and the aesthetic experiences associated with many art forms seem to lack the “typical” relation to survival functions (Juslin and Västfjäll, 2008) that has been the back bone of utilitarian emotion science (Ekman et al., 1983). This has led some authors to conclude that music does not induce “real-life” emotional responses (Konečni, 2008), or at least not survival driven (basic) emotions (Kivy, 1989). However, much of the evidence suggests that music can induce a huge variety of emotions (Zentner et al., 2008; Juslin et al., 2011; Coutinho and Scherer, 2017), and that these emotions have an evolutionary underpinning (Juslin and Laukka, 2003; Céspedes-Guevara and Eerola, 2018). What is certain is there is something different about music induced emotional experiences, or at least art induced emotions (Schindler et al., 2017), and a desire to explain these has led to the development of music specific emotion models. Yet, as we shall see in the CODA model we present later it is completely possible to group musical emotional experiences around the general mechanisms of emotion, moreover the mechanisms of general cognition.

We will now explore two popular models in music and emotion science (Juslin and Västfjäll, 2008; Juslin, 2019, BRECVEMA) and (Scherer and Coutinho, 2013, Multifactorial approach) that have guided much of the theoretical and methodological thinking in the field over recent years. These two models are chosen specifically because Juslin's model represents the most cited music emotion model and one that relegates the role of appraisal to a mediating mechanism in specific circumstances. In comparison Scherer and Coutinho's model represents the most comprehensive attempt to incorporate appraisal into musically induced emotions. Nevertheless, there are of course several notable models that have contributed substantially to scientific understanding (Robinson, 2005; Konečni, 2008; Flaig and Large, 2014; Koelsch et al., 2015). A recent musical adaption (Céspedes-Guevara, 2021) of Barrett's (2006, 2017) constructionist model is also explored in the Supplementary Material. We identify domains of overlap and isolate problematic areas within these theoretical constructs. We will show how appraisal has been incorporated into these models, both explicitly and implicitly, but to a large degree remains subordinate to other mechanisms. In doing so, we argue from a skeptical perspective that re-conceptualizing the importance of appraisals in current models is key to understanding musical emotions where the emphasis upon a stimulus' relevance and meaning to an individual is acknowledged.

Before beginning, it is fair to acknowledge that all models discussed have proved highly popular for capturing emotions in lab situations but also to some extent in real and even cross-cultural settings. However, the importance of acknowledging individual and situational differences is a recurring theme in meta-analyses and reviews (Eerola and Vuoskoski, 2013; Céspedes-Guevara and Eerola, 2018; Warrenburg, 2020). It is a task well suited to appraisal theories that highlight goal-directed relevance and meaning.

### 3.1. Emotion Induction Mechanisms for Music (BRECVEMA)

Juslin and Västfjäll (2008) proposed a set of eight mechanisms for induction of emotions via music, which included *brain stem reflex, rhythmic entrainment, evaluative conditioning, emotional contagion, visual imagery, episodic memory*, and *musical expectancy*. Juslin (2013) later added *aesthetic judgment* to the mechanisms, dubbed as BRECVEMA (initials from mechanisms). Each mechanism has a separate descriptive process referencing underlying brain areas, survival value, information focus, possible onset during ontogenetic development and the degree of dependence on culture and learning. Mechanisms differ in their availability to consciousness and induction speed. The central mechanisms such as contagion, brain stem reflex, episodic memory, and musical expectancy have been empirically explored (Juslin et al., 2014, 2015) leading to differentiated emotional responses, both using self-reports and physiology. The mechanisms have been used in numerous studies to eliminate unwanted triggers of emotions (such as memories or conditioned responses). However, the framework has been criticized for lack of specificity for mechanisms such as contagion (Madison, 2008; Malmgren, 2008; Thompson and Coltheart, 2008); the unacknowledgement of musical functions in process (Madison, 2008); convoluting utilitarian and aesthetic emotions (Moors and Kuppens, 2008; Scherer and Zentner, 2008); the fundamental role of appraisal in other emotion theories (Konečni, 2008); the lack of music as an “intentional object” (Robinson, 2008); issues between perceived and felt emotions (Thompson and Coltheart, 2008) and for its applications to contextual and cross-cultural understandings of music-emotion (Trehub, 2008). What interestingly unites all these commentaries is their explicit mention of appraisal in the music-emotion process.

This wide consensus likely emerges because Juslin and Västfjäll (Juslin and Västfjäll, 2008) make no use of cognitive appraisals in their model of musical emotions. In fact, as Madison (2008) and Holochwost and Izard (2008) note, they actively seek to remove cognitive appraisal from the process, representing appraisal as the philosophical “straw man” (Madison, 2008). Yet, as we note in our skeptical review, appraisal appears fundamentally necessary to the emotion process and has been clearly acknowledged in the music-emotion literature (Scherer and Coutinho, 2013; Eerola, 2017; Herbert and Dibben, 2018; Céspedes-Guevara, 2021).

Clear overlap between Juslin's mechanisms (Juslin and Västfjäll, 2008) and appraisal are evident. Here we follow Céspedes-Guevara (2021) in documenting where these overlaps exist. Low-level cognitive processes (*brainstem reflexes*) can be thought of as *novelty* appraisal check, associated with orientation (Eerola, 2017). *Musical expectancy* (Huron, 2006) can be seen as equivalent to *intrinsic pleasantness* (Céspedes-Guevara, 2021). Additionally, given the emphasis Huron (2006, 2019) places upon “mere exposure” and implicit learning in musical expectancy—simply explained as, familiarity breeds liking and can occur without conscious effort—it seems likely *familiarity* would also be highly relevant. It is important to specify that, if mere exposure and implicit learning influence intrinsic pleasantness, a degree of cultural variation should be observable, assuming that the mechanisms themselves are inter-culturally reliable. Evidence for the cross-cultural reliability of low-level BRECVEMA mechanisms (Barradas, 2017) and appraisals (Fontaine et al., 2013; Nordström et al., 2017) exists but appraisal has not been explored in a cross-cultural music context.

Juslin's (2019) most recent model suggests that cognitive appraisal is part of musical emotions but only at very low levels (e.g., brain-stem reflexes, contagion) where the *goal* is within the organism's design (e.g., protection, social understanding); distinct from high-level cognitive goals involving plans and motivations. There are examples where music induces an emotion by manipulating an organism's goals (e.g., loud music while trying to study). However, Juslin counters that these do not represent the typically induced musical emotions—2% according to an experience sampling study by Juslin et al. (2008; in Juslin, 2019)—and do not place the emotion within the music like other mechanisms. Juslin therefore includes goal-relevance in his most recent works but suggests that although a goal may be present (e.g., relaxation) it is usually not the mechanism that causes the emotion. For example, *Jane's goal is to relax. She plays her favorite song and relaxing feelings are induced by the visual imagery mechanism*. The goal is then achieved but is not the cause of the emotion. Adopting this position suggests goal-relevance can become a mediating mechanism of higher cognitive processes but other mechanisms are likely to determine most of the variance in emotional processes. Juslin contends that without goal-relevance almost all mechanisms could be seen as appraisals. Presumably, the problem being this forces together very distinct mechanisms (e.g., visual imagery and entrainment).

Nonetheless, a diverse range of sources acknowledge the ways that people use music for goal-achievement; *distraction, energizing*, and *mood enhancement* (Sloboda and Juslin, 2010) and *social bonding* (Clayton, 2017). Van-Goethem (2010) noted that *mood-regulation*, part of the umbrella *mood-enhancement* motivations, accounted for over half of people's listening motives. Juslin would possibly argue as above that these outcomes are achieved through alternative mechanisms. However, skeptical theories would rebut this because, without goals to explain (musical) emotions the emphasis falls wholly upon the music. Goals are fundamental to general cognition and the ongoing interaction between organism and environment.

Consider again Jane's experience. This time, in the same physical context (at home, alone) and with the same music *Jane has the goal to set a romantic scene for an impending date; Jane produces visual images of moonlight walks along the beach and feels excited*. In another example, *Jane is upset that her date has canceled. Playing the same music elicits comfort and acceptance*, possibly with a mediating mechanism like visual imagery or another mediator, like episodic memory. What is clear in these examples, is that Jane's goals in different scenarios have changed her emotional experience of the same piece of music in the same context. Furthermore, different goals can be seen to lead to the activation of other mediating mechanisms and behaviors in an emotional episode. Importantly, from a skeptical viewpoint these mechanisms utilize existing non-emotional cognitive mechanisms to produce the most valuable behaviors for achieving one's goals. In moving the dependent variable from the diverse number of discrete emotions that Jane may have experienced in any of these scenarios to musical behaviors (e.g., moving in time) or conscious experience (e.g., visual imagery) we can explore the relationship between emotional components to assess how the utility of a particular behavior allows it to take precedence over other possible behaviors through goal-directed mechanisms.

Juslin (2019) extends his discussion on “aesthetic judgement” a mechanism commonly described through appraisal (Egermann and Reuben, 2020). Aesthetic and utilitarian emotion are distinguished by this underlying mechanism. Simply, aesthetic judgments produce aesthetic emotions and all other mechanisms do not. Two types of affective responses are possible, “preference for” and “emotion specific.” Juslin states in the absence of any other mechanism “preference … and aesthetic judgment will be consistent with each other” (Juslin, 2019). Aesthetic judgment can also differ from preference, underpinning Juslin's explanation of all mixed-emotional responses. For example, *Jane experiences sadness through episodic memory, while aesthetic judgments produce feelings of beauty*. Juslin suggests “conditioning” and “contagion,” as more implicit mechanisms, may be immune to such effects from aesthetic judgments. The suggestion therefore, that aesthetic emotions account for mixed emotions (e.g., guilty-pleasures) or positive associations with sad music becomes problematic. If an aesthetic response (beauty) can override the contagion of sadness, the prediction that low-level mechanisms are more robust to the influence of aesthetic judgments is undermined. In a similar vein to Scruton (1999), Juslin notes that aesthetic emotions can be negative. He believes these negative aesthetic responses mix with other mechanisms to create so called “guilty pleasures.” Yet, no explanation of how different mechanisms interact, take precedence, by what degree or in what circumstances is offered.

Aesthetic judgments take a privileged position in Juslin's model through their unidirectional influence upon other mechanisms. Furthermore, assuming that most musical emotions are to some degree aesthetic, means this mechanism should be active in almost all examples and be the default music-emotion mechanism, while other mechanisms become predominantly mediating variables in different contexts. This is difficult to justify in a theory that takes such a strong evolutionary stance (Juslin and Laukka, 2003) but offers no theoretical argument for the evolutionary development of aesthetic judgments as superior to other evolved mechanisms. Skeptically, one would argue that evolutionary evolved appraisals, are better understood through an individual's goals. Instead of aesthetic appraisals, aesthetic goals like distraction (Saarikallio et al., 2020a), or meaning enhancement (Sloboda and Juslin, 2010), become the studied phenomena while the aesthetic terms Juslin ascribes to aesthetic appraisal (e.g., beauty) become folk terms relevant only to the socio-cultural environments in which they emerge.

### 3.2. An Alternative Multifactorial Process Approach

An alternative appraisal focused interpretation of emotional induction mechanisms for music was put forward by Scherer and Coutinho (2013). This theory critiqued the BRECVEMA (Juslin and Västfjäll, 2008) suggesting many of Juslin and Västfäll's proposed routes can be subsumed under appraisal, as discussed above from Céspedes-Guevara's (2021) perspective. Most notably in Scherer and Coutinho's critique of BRECVEMA is the lack of distinction between levels of function (Marr, 1982; Bechtel and Shagrir, 2015) or discussion on their interaction. Instead, Scherer and Coutinho (2013) expanded on previous work (Scherer and Zentner, 2001) that emphasizes the multiplicative interaction of structural, performance, listener, and contextual features (“production rules”). Within these factors they propose five routes that can lead from music's structural features to fuzzy-set emotions via the mediating factors of listener, performer, and context. These routes include memory, entrainment, contagion, empathy, and appraisal. We will focus here specifically on the appraisal route as it is the main focus of the article and because many of these mechanisms have been covered in Juslin's (2019) BRECVEMA. It is however worth noting a few key distinctions in some mechanisms. Empathy forms a significant expansion upon the contagion mechanisms, which can be placed solely within expressive cues. Empathy instead must include an understanding of another's motivations and appraisals of a situation (p. 139). Moreover, entrainment while seen as separate to memory and appraisal systems is suggested to have an influence upon and be influenced by these other systems through “disinhibition” (“facilitation of preexisting emotions”).

Appraisal forms a key component in this interaction. It evaluates the structural features of the music (both psychophysiological and macro level features) for their relevance to a listener. Appraisal further drives and coordinates the sub-components of emotion and can be both an automatic and “effortful” process. The appraisals mechanisms hypothesized are grouped around their broader functions including relevance to the organism, implications, coping potential, and normative-significance (cultural norms and personal values). They discuss examples for each appraisal mechanism in relation to how the appraisal can be linked to musical features but also describe several examples in relation to musical activities. It is noted that because of the different goals that musical and non-musical situations may provide, different appraisals may be more or less present in musical situations. According to Scherer and Coutinho appraisal cannot alone account for the emotional experience of music but appraisal should be included. They propose that appraisal processes allow for the distinction between fuzzy-set emotion categories (utilitarian, aesthetic, and epistemic) along with patterns across other emotion components. Music emotions are subsumed under the categories of aesthetic and epistemic emotions though blending between categories is possible.

A skeptical perspective suggests several benefits that are incorporated by Scherer and Coutinho (2013) account. Specifically, a more inclusive account of appraisal's role within the music emotion process allows the musical mechanisms to be linked with those of affective processing in general. Furthermore, appraisal allows for the incorporation of the wider context in which musically induced emotions occur. However, while Scherer and Coutinho's approach draws heavily from a specific framework within the skeptical perspective (Scherer, 2009a,b), three core critiques should be acknowledged from the wider collection of skeptical theories that should be noted.

First, Scherer and Coutinho (2013) are critical of the basic emotion approach and claim that discrete emotions should not be the active point of study. We adopt this stance in the current proposal as well. Scherer and Coutinho sought to solve the problem by proposing their own “fuzzy-set” categories (utilitarian, aesthetic, epistemic). However, this again leads the conversation back to how the emotion space is organized instead of focusing on the emotional episode and its mechanisms.

Second, Scherer and Coutinho note the importance of appraisal in coordinating other sub-components of the emotion process through their interactive nature. However, a focus toward the end goal of a specific emotion (discrete or fuzzy-set) restrains the analysis of this process to a linear operation. One that leads directly from input (musical stimulus) to output (fuzzy-set emotion). The wider skeptical approach instead seeks to study the dynamic and recursive emotion process. This focuses the empirical lens toward the interaction between emotion sub-components over time.

Third, Scherer and Coutinho emphasize the importance of goal-conduciveness within the music emotion process. Their approach is again situated in distinctions within the emotion space and draws heavily on Kant's (2001) perspective of “disinterested pleasure” (this being defined as relevant to aesthetic and epistemic concerns but more closely aligned with a lack of goals). However, understanding aesthetics through the lense of “disinterested pleasure” does not sit well in the current biological science (Huron, 2019). To situate music in a context, an ultimate goal of appraisal theory, Moors et al. (2017) notes that the appraisal process is better understood through the competition of multiple competing goals, an approach that allows greater individual variation within the appraisal framework. Moreover, recent developments in embodied and enactive goal-directed accounts of meaning construction suggest goals are an active part of our musical lives (Schiavio et al., 2017b). The diverse and well documented reasons people have for engaging with music (Saarikallio, 2011; Schäfer et al., 2013; Randall and Rickard, 2017) can be constructed around the affordances of the acoustic environment (discussion and examples in CODA model).

Finally, though not explicitly related to skeptical perspectives, the authors note that the explicit focus on the cognitive aspects of the emotion process does not allow for a broader perspective of how different levels of analysis may fit together. This is an approach that Eerola (2017) has previously mapped between the BRECVEMA and constructionist models and one we seek to expand on in our model with the incorporation of dimensional-appraisal. Other notable attempts to integrate the constructionist model have been made. Julian Céspedes-Guevara (2021) outlined his interpretation which incorporates some aspects of appraisal theory, but is more influenced by Barrett's (2006, 2017) adaption of constructionist theories focusing on categorization. Barrett states that “valuation” (meaning appraisal; “organisms continually and automatically evaluate situations and objects”) and the “conceptual act” (the segregation of core-affect into different states through linguistically categorizing sensorimotor information) are key to understanding emotion. Yet, little is said about appraisal beyond its consistency with her notion of contextual sensitivity “the fundamental assumption of appraisal views: the meaning of a situation to a particular person at a particular point in time is related to the emotion that is experienced.” (p. 33). This is closest conceptually to Clore and Ortony's (2000) conception of appraisal where categorization is supported by mechanisms that allow for adaptive goal differentiation. Céspedes-Guevara's (2021) model is described in full and evaluated from a skeptical perspective in the Supplementary Material.

## 4. The CODA Model

Here, we present our skeptically-informed constructionist-appraisal model. Ultimately, the goal of this model and the discussion of its hypotheses is placed upon explaining the emotional experience of the listener. Nevertheless, we hope it provides the scope for expansion where other perspectives (e.g., performer or composer) can be considered. First, we re-evaluate the goal-directed role of *dimensional*^4^ appraisal in a musically induced emotional episode. We establish the value of dimensional appraisal in the ongoing construction of relevance and (musical) meaning to a listener, as well as the dynamic, weighted and bidirectional relationship appraisal has with other multidimensional components of emotion as an individual interacts with the (musical) world. In the discussion of this model, we highlight its contribution to resolving existing gaps in the literature through specific hypotheses and implications for future research. In conclusion, we point to how these ideas will allow researchers to ask different questions about the nature of emotion, ones that explore the continuous generation of relevance and meaning of a stimulus to an individual.

### 4.1. Building on What Exists

This model is intended to build on previous research. We have demonstrated that substantial gaps in existing music-emotion models remain. To help resolve them, we use evidence from skeptical theories to extend current understanding of emotional episodes induced by music. Skeptical theories counterpoint the limited adaptions of discrete theories postulated by the existing music-emotion models discussed in this paper (Juslin, 2019). Furthermore, we have shown how discrete approaches, with a predominant focus upon differences within the stimulus (e.g., acoustic cues and their relationship to discrete emotions) have generated an ever increasing number of mediating mechanisms to be incorporated, thus leading to reductive theories.

Alternative music emotion models (Scherer and Coutinho, 2013; Céspedes-Guevara, 2021) favor more skeptical notions. Scherer and Coutinho (2013) explore the role of appraisal while Céspedes-Guevara (2021) has moved the field closer to constructionist accounts such as Barrett's (2017) adaption of PC. Such ideas have been invaluable in showcasing the need for identifying new methodologies (Céspedes-Guevara and Eerola, 2018) beyond stimulus-driven approaches. However, the continued focus on categorization has hindered the nuanced role of relevance and meaning to an individual in music emotion models. A process dimensional-appraisal (dynamic cognitive evaluations) is well adapted for, when placed within a larger framework that acknowledges other levels of analysis (Eerola, 2017).

### 4.2. Key Elements: the CODA Model

**Figure 1:**
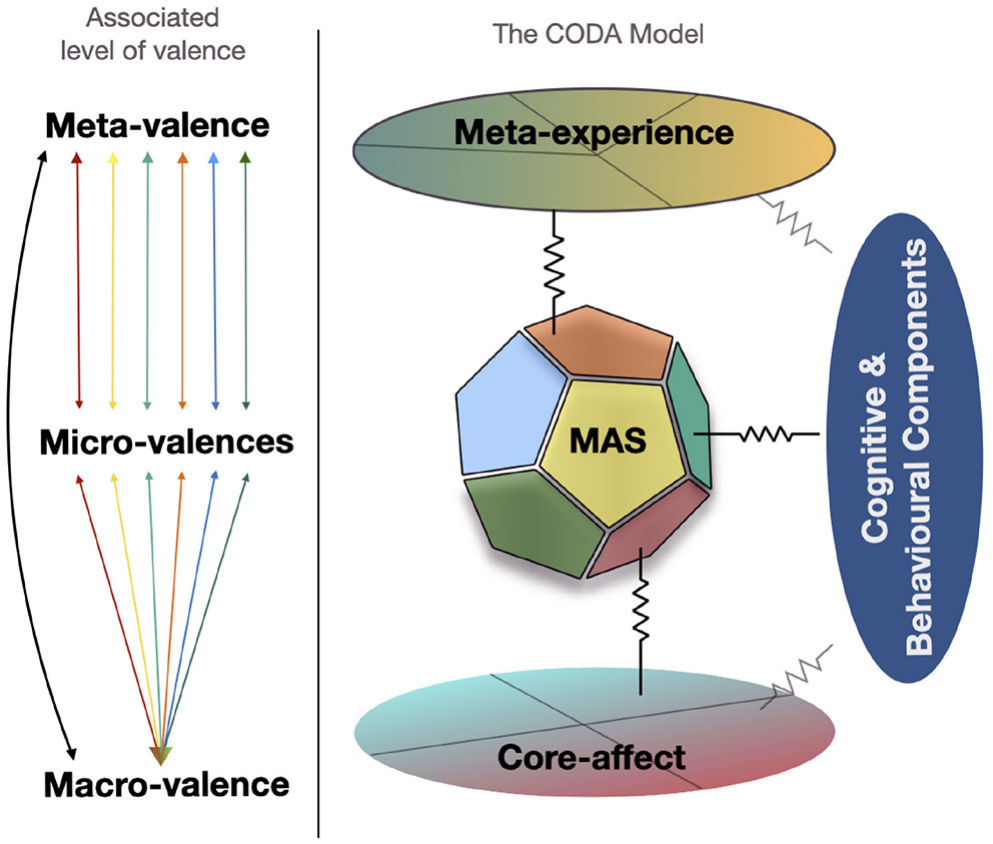
Schematic outline of the CODA model with the associated levels of valence for each dimensional component of the model.

Here, we summarize our new model the *Constructivistly-Organized Dimensional-Appraisal* model - the CODA model (Figure 1). Our model stresses the cyclical and interactive nature of appraisal within emotion process; hence its name referencing the concept of “once more from the beginning.” From a skeptical perspective, we build on top of dimensional constructionist accounts of emotions offering a concise framework which not only captures emotional processes but frames it within the mechanisms of general cognition in a dynamic structure that recursively re-informs itself.

This section will outline the key elements of our model including **core-affect**, **appraisals** (multidimensional appraisal space, appraisal dynamics, their bi-directional and weighted nature), **component interactions** over time, and finally **meta-experience**. The discussion of each of these elements is framed within the ongoing construction of relevance and meaning that occurs between an organism and its environment.

#### 4.2.1. Core Affect

*Core affect* refers to the neurophysiological state described by Russell (2003, 2009, 2012) where changes in core affect drive attention through the process of attributing these changes to a stimulus. Changes in core-affect in music are typically linked with psychoacoustic cues (Céspedes-Guevara and Eerola, 2018). This meta-analysis rebuts previous attempts to link acoustic cues to “folk” terms (Juslin and Laukka, 2003). Instead, they show the foundations of musical expression in vocalizations is predominantly correlated with changes in arousal. Valence however, produced significantly fewer unambiguous patterns that map to psychoacoustic cues, coinciding with findings that valence is expressed differently in different genres (Eerola, 2011). Overall, evidence points toward an understanding of core-affect that is beyond reductive psychoacoustic mappings to physiological states.

To explain such meaning construction we invoke embodied and enactive accounts of music, suggested by multiple authors (Clarke, 2005; Reybrouck and Eerola, 2017; Schiavio et al., 2017a)—i.e., goal-directed. A goal-directed dimensional-appraisal account of cognition (see Appraisals) allows for a more dynamic, embodied and enactive conception of core-affect's interaction with acoustic cues for an individual in a situational context and identifies several mechanisms that inform the construction of valence.

One of the most common critiques of core-affect is its inability to represent mixed emotions. This is an important caveat for a theory of musically induced emotions where mixed emotions are commonly acknowledged (Hunter et al., 2008, 2010). It has been postulated that mixed emotions are captured by core-affect as a rapid switching between different activities in a dimension (Eerola, 2017; Russell, 2017b) this activity linked with perceiving multiple *affective qualities*^5^ of a stimulus with different valences (e.g., appreciation of the composer but not the performer) co-occurring with changes in core-affect. Appraisal models compatible with this idea have also been put forward. One that additionally seeks to differentiate the intensity of mixed emotional experiences is Roseman (2017).

#### 4.2.2. Appraisals

In our skeptical critique of two models of musical emotions, we argue explicitly for a stronger and more nuanced incorporation of goal-directed appraisal. Here we outline explicitly what this would entail. We describe the multidimensional nature of appraisal space, the individual dimensions of this multidimensional space, these dimension's micro-valenced nature and parallel processing, and finally, the weighted contribution of appraisal dimensions to other components of an emotional episode.

##### 4.2.2.1. Multidimensional Appraisal Space (MAS)

The polyhedron at the center of our model (Figure 1), represents multidimensional appraisal space (MAS). We place MAS as a key cognitive-affective component in the construction of *relevance* and *meaning* for an individual. MAS consists of *n*-dimensions (appraisals). MAS can be seen as a dynamic and ongoing perceptual interaction between a person and their physical and social environment. The interaction between MAS and other cognitive components can be seen as a weighted probability function of each appraisal dimension dependent on the interaction between external and internal factors. As the interaction between external and internal conditions develops, the weighted nature of any appraisal can change (i.e., their non-linear nature). This weighted interaction between MAS and other components is discussed in terms of individual dimensions. Thus, between two consecutive time steps the probability of an appraisal producing the same result is not fixed. Simply, changes in any single dimension will lead to changes in MAS and consequently the continuous development of individual *shades of meaning*.

MAS can evaluate multiple types of information, verbal-like, sensory, perceptual and symbolic. These evaluations can be both *rule-based* (active computation—but still typically unconscious) and *associative* (learned or memory-based associations between a stimulus and an appraisal output). The output from MAS represents graded distinctions between rule-based and associative processing where each appraisal can occur in a rule-based or associative way. This graded distinction can change between time-steps.

For an example, we return again to Jane. *Jane's first experience of heavy-metal music comes with expectation that dancing would accompany live music (associative processing) but the novelty of the type of behavior (moshing) that emerges and the conduciveness of using such behavior for enjoyment are rule-based processes*. The output of MAS can then be seen as a graded distinction between more or less associative processing. This example can be further expanded beyond what music affords to show how graded distinctions of processing can occur within music too. *Jane may be unfamiliar with the timbral structure of the new music (e.g., more dissonant chord progressions) and process this in a more rule-based fashion. The rhythm however (fast and regular), remains similar to other stimuli Jane has heard and can be processed in an associative way*. Such examples can be easily extended to cross-cultural perceptions of music.

In constructing MAS we align our thoughts with others (Scherer, 2009b; Schiavio et al., 2017a) who highlight the importance of new non-regressive methods such as dynamic system theory (DST) and other time series analyses in capturing the ongoing and bi-directional nature of an individual's interaction with the world, including its sonic environment.

##### 4.2.2.2. Dimensions of MAS (Appraisal Dimensions)

**Figure 2:**
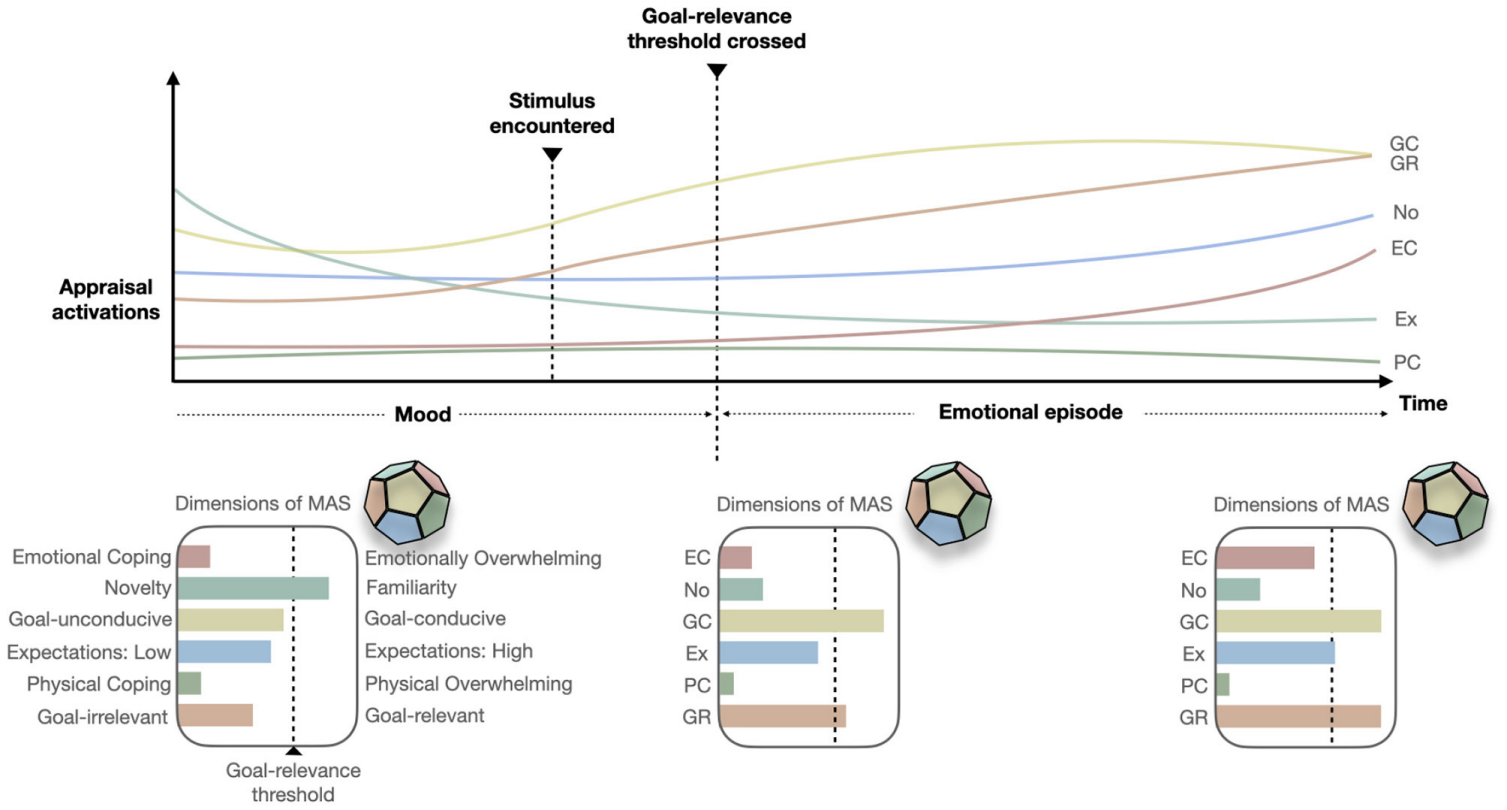
A visual representation of the dynamic changes in appraisal dimensions of the multidimensional appraisal space (MAS) in the CODA model. Only a few key appraisals are plotted for illustrative purposes. MAS is truly representative of *n* dimensions. The timeline shows a hypothetical encounter with a stimulus the drives changes in appraisals. As the goal-relevance appraisal crosses a threshold general cognitive processes become a subjectively perceived emotional episode. The plots below the timeline offer snapshots of MAS at different time stamps and highlight the activation of each appraisal dimension. Note that the extremes of each dimension are not clearly positive or negative. Appraisal dimensions code: GR, Goal-relevance; GC, Goal-conduciveness; No, Novelty; Ex, Expectations; EC, Emotional coping; PC, Physical coping.

Appraisals represent the dimensions of MAS (Figure 2). This does not represent a comprehensive list of all appraisals. There are numerous theories (Oatley and Johnson-Laird, 1987; Clore and Ortony, 2000; Scherer, 2009b; Ellsworth, 2013; Frijda, 2017) that predict varying numbers and types of appraisals. Our model is conservative in discussing appraisals with significant agreement across theories (Moors et al., 2013) and shows evidence for an underlying neurological structure (Brosch and Sander, 2013); though additional dimensions are possible.

*Novelty*, (familiarity) is key to musical understanding (Meyer, 1956; Huron, 2006) and has an underlying neurological structure (Brosch and Sander, 2013; Kafkas and Montaldi, 2014). Neurologically novelty is a dual-route system. Independent systems (novelty and familiarity) converge and interact in fronto-parietal areas forming an assessment of “relative-familiarity” (Kafkas and Montaldi, 2014), which cannot be deconstructed at a psychological level. Novelty is highly influenced by previous musical exposure and is continually reinformed by *expectations*. Neurologically this system is tied to memory circuits which use novelty and familiarity signals heuristically (Kafkas and Montaldi, 2014—see micro-valences for heuristic processing; Gigerenzer and Gaissmaier, 2011). Novelty plays a key role in attention orientation, along with *goal-relevance*; both linked with processing in fronto-parietal areas key in information integration (Balleine and Dickinson, 1998; Brosch et al., 2013; Kafkas and Montaldi, 2014). Novelty appraisals can assess situational and sensory information types but remains, in many forms, an unexplored element of emotional episodes induced by music (see affordances in *goal-relevance* and hypotheses).

*Expectations*, like novelty, is one of the first musical appraisals to be linked with meaning generation (Meyer, 1956; Huron, 2006), tied to melodic and rhythmic musical elements cross-culturally (Mehr et al., 2019) and guided by novelty toward heuristic access of existing schema. Expectations are also informed by music's functional, situation and extra-musical components. For example, extra-musical expectation is violated in musical fusions like the use of a didgeridoo in a western orchestra playing an interpretation of the Australian pop tune “Down Under” (Australia, 2019). There is evidence that musical functions can be perceived across cultures too (Mehr et al., 2019), noting that systematic, but probabilistic, variance in acoustic features are predictive of functions (dance, healing, lullaby, and love songs) at above change levels. This does not imply pre-determined biological responses but instead a close link between acoustic cues and affordance (see *goal-relevance*).

*Goal-conduciveness* represents an evaluation of how conducive/obstructive an event is to an individual's current goals. It is closely tied to the type of goal a listener has (e.g., choosing to listen to music and hearing music without explicit choice) and the situation in which a listener encounters the music (e.g., a noisy environment where a chosen piece of music cannot be heard). It can equally be applied to sonic goals and the conduciveness of the music (e.g., a lack of tonal complexity when searching for new musical insights (Gabrielsson, 2011).

*Certainty* is an individual's assessment of the likelihood of a perceived outcome, be it acoustic, functional, situational, or extra-musical. This dimension is likely to become more significantly weighted in relation to other appraisal dimensions as the degree of certainty decreases, closely tied to familiarity. For instance, the certainty of enjoyment in a live show is reduced if it is too crowded or loud. Certainty can be applied to the music too, for example chord progressions in an unfamiliar pop song. Note that such an appraisal incorporates extra-musical knowledge of the formulaic nature of pop music. Certainty can play a key role in prioritizing competing goals (i.e., prioritizing a less important goal over a more relevant one because the likelihood of a positive outcome is more certain).

*Coping-potential* can be both physical (e.g., the ability to remove oneself from a deafening loudspeaker) and emotional (e.g., suddenly hearing a song you associate with a loss). We highlight the variety of types of information coping-potential can appraise (not only verbal-like). Others have considered the term in aesthetic ways. Silvia (2005a,b); Silvia (2006) describes coping potential as “ability-to-understand.” He has shown how this appraisal, along with novelty, is key to aesthetic experience. One can see how an individual's ability to follow the progression of a song or tap along with the pulse can change the emotional experience and behaviors (e.g.,dancing) they produce.

It is interesting to consider that a lack of emotional coping-potential in a musical context is not always negative. In many examples feeling emotionally overwhelmed is described as positive (Gabrielsson, 2011, ch. 6) and prized for its uniqueness. Strong emotional experiences (i.e., highly *goal-relevant*) can lead to the development of new goals (affordances) (Gabrielsson, 2011, ch. 15) such as seeking out a recording of the track or listening to it repeatedly (aesthetic) or music as an aid for sleep (utilitarian).

*Agency*, in a musical context refers to the composer, a performer, or oneself (Scherer and Zentner, 2001). For example, a listener may attribute a strong emotional response to a song they have heard many times before to the way a particular performer plays it, or to the sonic quality of the environment. Alternatively, the lack of emotional engagement with a well-known and liked song may be attributed to one's existing state (e.g., “*I'm* just not in the mood for this type of music right now”—less goal-relevant).

*Goal-relevance* is an evaluation of the utility (i.e., the value) of different *affordances* (goal-directed actions). *Affordance* in this instance refers to cognitive processes of evaluating the utility of different actions (including mental actions) that different stimuli offer, as opposed to the stimulus-driven affordances proposed by Gibson (1977). Thus, we imply an *embodied* and *enactive* account of cognition similar to affordance in 4E cognition (see Newen et al., 2018) where goal-directed action is fundamental to a dynamic system of meaning construction. We associate affordances closely with the many motivations people have for engaging with music and functions music serves, noted in several chapters of multiple handbooks (Juslin and Sloboda, 2010; Hallam et al., 2016; see Maloney, 2019 for a review). These can include desires, beliefs, wishes, needs. Yet, the number and type of goals associated with music could be endless. We distinguish, as Alan Merriam did (1964), between the many uses of music and its functions (the broader reasons and purposes it serves). Musical affordances can represent both innate evolutionary concepts (Madison, 2008) as well as personal, social, and socio-culturally constructed goals (Boer et al., 2012), including aesthetic goals (Moors and Kuppens, 2008; Menninghaus et al., 2019; Zickfeld et al., 2019). Moreover, affordances can encompass the associative and symbolic meaning attached to music (e.g., the lyrical narrative or the mimicry of bodily motions). Evidence for the link between goals and affordances can be seen in Schäfer et al. (2012, 2013) linking musical functions to the formations of musical preferences.

Goal-relevance, like *novelty*, is closely tied to attention and physiological orientation (Brosch et al., 2013; Scherer and Moors, 2019; see also Nieuwenhuis et al., 2011 for the link between goal-relevance, P300 event-related potential, and increased cognitive and behavioral “responsivity”). In line with Moors et al. (2017), we propose that a goal-relevance threshold marks the distinction between emotional and non-emotional cognition as a graded continuum upon which individuals differ. Goals can be processed subconsciously, and often represent multiple competing goals. Cybernetic models have similarly been designed around multiple competing goals directing cognitive control and behavior (Inzlicht et al., 2015). Contextual modifiers are an important source of information in distinguishing goals, where different situations may afford new goals that take precedence over existing ones. Furthermore, the types of goals that are prioritized more frequently may differ both individually and cross-culturally (Saarikallio et al., 2020a).

The importance of a goal-directed system in the construction of musical behaviors and meaning has been highlighted in other theoretical work on musical development. “Teleomusicality” (teleo from Greek - goal, Schiavio et al., 2017b) suggests how music specific (sound oriented) goals become apparent in infants and develop in complexity. We too note that “musical actions (including listening) are always motivated (goal-directed)” (Schiavio et al., 2017a, brackets in original). The dynamic and non-linear construction between embodied perception and action in shaping this is paramount to individual musical meaning attribution. However, we expand on the concept by allowing for a dimensional, rule-based, and associative interpretation of *goal-relevance*, allowing greater flexibility in the way ongoing meaning construction can be modeled and furthermore offer testable hypotheses.

##### 4.2.2.3. Appraisals as Micro-Valences

Here we suggest that the dimensions of MAS (appraisals) can be viewed as “micro-valences” (Shuman et al., 2013). Valence distinctions are an important contribution of our model (left side; Figure 1). Different levels of valence are associated with different levels of the CODA model and therefore different levels of processing. Each appraisal dimension represents a micro-valence. That is each appraisal dimension makes a qualitatively different valenced contribution to subsequent processes. For instance, the novelty-familiarity appraisal dimensions can be seen as a form of valence, where too much or too little novelty is negatively processed but an appropriate amount of novelty is positive (Berlyne, 1971). The valenced contribution of individual appraisal dimensions can interact bidirectionally with macro-valence (valence in core-affect) and meta-valence (the emergence of macro and micro-valences in consciousness). These valences are ongoing processes, in line with the cognitive components associated with them (appraisal dimensions). By incorporating micro-valence with one-dimensional macro-valence we counter several critiques of one-dimensional valence (Shuman et al., 2013) that have been applied to Russell's (Russell, 2003) model (see Psychological Construction).

Evidence for this dynamic and bidirectional interaction comes from Kuppens et al. (2012). They demonstrated how several appraisal dimensions influence core-affect, and vice-versa, in everyday life. Individual differences in the strength of these relationships were also noted, suggesting that individuals, situations, and stimuli can influence the way appraisals interact with macro-valence. Shuman et al. (2013) suggest that micro-valences interact with macro-valence as the product of a weighted sum. Variability in the relevance of micro-valences suggests changeable weightings in different circumstances. Evidence for such heuristic information processing comes from Gigerenzer and Gaissmaier (2011) who note *recognition* (memory informed appraisal bias, where similar appraisal outputs to similar stimuli weight certain appraisals more highly), *one-clever-cue* (identifying the most salient cue), and *trade off* heuristics (equal weighting of appraisals). Which process takes priority is informed through *ecological rationality* where in different situations “with sufficient experience, people learn to select proper heuristics from their adaptive toolbox” (Gigerenzer and Gaissmaier, 2011). In other words, a goal-directed interpretation of heuristic selection—the heuristic with the most utility to achieve one's goals (see Gigerenzer, 2021 for an example of goal-directed *embodied heuristics*). Heuristic processes can lead to different contributions of negative and positive appraisal weightings to other components. For instance, a piece of music with too much melodic novelty in an otherwise agreeably appraised piece may influence an individual's overall macro-valence more negatively.

Individual differences are evident in these processes too. Evidence for both *positivity offset* & *negativity bias* has emerged (Cacioppo et al., 1999). Individuals with greater positivity offset may develop a liking for a neutral stimulus (e.g., a new song) after fewer exposures. The strength of these biases is relatively stable across individuals (Ito and Cacioppo, 2005). Other research suggests individuals may systematically differ in appraisal weightings, such as optimistic and pessimistic individuals' ratings of certainty and control (Lerner and Keltner, 2001, study 2) or sensation seeking individuals (Shuman et al., 2013). Such types of inherent *appraisal bias* have also been noted at a cultural level (Scherer and Brosch, 2009).

Micro-valences may also influence micro-valences at later time steps (Shuman et al., 2013), for instance, more effective processing of congruent information. Certain appraisal outcomes may constrain other appraisal outcomes (e.g., an appropriate degree of novelty may lead more naturally to a positive appraisal of coping potential—*ability to understand* Silvia, 2005a). Finally, appraisal outcomes can carry across situations. Evidence for this comes from Bechtel and Shagrir (2015, study 4) who show certainty and control appraisals influenced judgments of risk perception in unassociated situations. However, more research is needed to understand how appraisals may constrain future appraisal outcomes and therefore how micro-valences may constrain future micro-valences. What is important to note is how wide-ranging the source of micro-valences can be. For example, hormones, diurnal cycles, the immune system, or drugs (Russell, 2009) can all play a role in shaping macro-valence. Diurnal cycles have been shown to influence music choice (Park et al., 2019). The contribution of each of these will be based on biological, individual, and situational factors yet remain clearly interpretable through a goal-directed interpretation.

Micro-valences may play a role in understanding mixed emotional responses to music. Indeed, Russell himself (2017b) notes that mixed emotions can be explained by incorporating multiple assessments of *affective quality*, a concept we and Russell link closely with appraisal^6^. Multiple aspects of an object or situation can be evaluated differently leading to mixed feelings (Huron, 2016). For example, a positive appraisal of the acoustic elements with a negative appraisal of the song's message, may lead to a guilty pleasure. The extent to which micro-valences contribute to mixed emotional experiences may vary considerably across individuals and situations. Moreover, meta-valence (below) may be another component that can produce mixed emotions, either through multiple emotion “scripts” emerging in consciousness (e.g., fear and excitement on a roller-coaster) or through different valences emerging in different emotion components as changes happen over different time scales (Russell, 2017b).

##### 4.2.2.4. Parallel Processing of Appraisals

The appraisal dimensions of MAS are ongoing and processed simultaneously. The hierarchical nature of appraisal processing (Scherer, 2009b), where certain appraisals are processed before others (novelty, goal-relevance) when a new stimulus is encountered, does not change the fact that all appraisals are ongoing. Thus, changes in one appraisal produces changes in MAS as a whole, even if other appraisals are yet to be processed. We remind the reader how appraisals may constrain each another. Furthermore, re-appraisal can occur as appraisals are processed and feedback from other components is received.

Appraisal dimensions can provide evaluations of multiple aspects of a situation (e.g., the music, the venue, the audience, the performers, musical functions) or a stimulus (e.g., the lyrics, individual instruments). The processing of multiple aspects is not simultaneous, though may appear so at a conscious level. Continued attention (guided by bottom-up and top-down processes, e.g., core-affect, novelty, goal-relevance) will likely lead to further appraisal of more aspects (i.e., facilitated by increased cognitive and/or physiological responsivity, Nieuwenhuis et al., 2011). These appraisals can change over time as the stimulus or situation develops allowing a greater focus on different aspects of an episode to be more or less prominent at any given moment.

Appraisal outputs can be stored in working memory incorporating multiple appraisal outputs (e.g.,the violin solo, the lyrics, the venue, the performance etc.). In this sense appraisal aspects can be conceptualised in more or less gestalt ways. Individual differences in these may emerge. For instance, a listener with greater musical experience may individually appraise sections of an orchestra or particular instruments more readily than the non-experienced listener. Huron (2016) has noted the importance of *plural pleasures* in aesthetic experience where multiple components of hedonic value (aspects of appraisal) are often combined for a better experience.

##### 4.2.2.5. Appraisal Weightings

Appraisal dimensions make a weighted contribution to other components. This idea links closely to recent developments in dimensional-appraisal theories that allow for more sophisticated and non-linear modeling techniques (Scherer, 2009b). We construct this idea around similar models of perception (e.g., the lens model, Brunswik, 1956) commonly cited within the perception of acoustic cues in music (Scherer, 1986; Juslin and Laukka, 2003). It appears logical, even actively beneficial, to propose that both the stimulus-driven and goal-driven sides of the model would behave in the same weighted (partly redundant) way. Moreover, we note how the weighted nature of appraisals can change over time. As noted in the above sections, the ongoing development and change in weightings may be closely tied to other appraisals and their bi-directional interaction with other components.

#### 4.2.3. Meta-Experience

As Russell describes, *emotional meta-experience* is the conscious experience of a specific emotion (2003). This categorization is based on other components of emotion (e.g., core-affect, appraisal, the antecedent event) and is formed around prototypes. No specific mechanism (e.g., brain-stem, contagion, etc.) is needed to account for any given “folk” term because they represent the coherent experience of an individual (Colombetti, 2014) and their construction of the concept (a discrete emotion). The explicit categorization of an emotion is not required in an emotional episode but may emerge. It is distinguished from a general *meta-experience*, which implies the conscious awareness of multiple components, in that one does not explicitly need language to consciously experience these components, nonetheless it can certainly include labeling.

Concerning levels of valence (left-side; Figure 1), we note that individual micro-valences are accessible to conscious meta-experience as well as macro-valence in the form of a single composite of core-affect. Note however, that core-affect is not separable at higher levels of processing. This is represented by the non-orthogonal crossing lines, suggesting as valence reaches extremes arousal automatically increases (Kuppens et al., 2017).

#### 4.2.4. Component Interactions

Our model contends that appraisals form a variably weighted bi-directional *dynamic interplay* with other cognitive components. This is represented by the resistors between components. The weighted interaction between different components depends on several factors. Some of these factors are likely to be biologically driven while other factors such as individual and contextual factors may also influence the strength of these component interactions through changes in appraisal. For instance, conceptual knowledge of a situation and music's functions within it as amenable to dancing, along with a pre-existing desire to dance (*goal-relevance*), appraising the *novelty* of the music as well-balanced with sufficient physical *coping potential* to keep up with the music may strengthen interactions with *action tendencies* and *motor components*. In this example it becomes evident how appraisal can shape continued meaning generation. Evidence for such interactions has emerged between appraisals and behaviors (Brosch and Sander, 2013), physiological responses (Aue and Scherer, 2008, 2011), facial expressions (Scherer et al., 2017), vocal expressions (Laukka and Elfenbein, 2012; Belyk and Brown, 2014; Nordström et al., 2017), and core-affect (Kuppens et al., 2012; Shuman et al., 2013). We note that the interaction between components can occur with minimal weighting to appraisals and have included these interactions too (right-side; Figure 1). However, we contend this route is a-typical of musical emotion experiences hence represented with a lighter shading.

#### 4.2.5. Concluding Remarks

Our model captures how appraisal facilitates a music-elicited emotional episode through the ongoing construction of relevance and meaning between an organism and their (musical) environment. It has been informed by skeptical theories of emotion that seek to move beyond the reductive construction of emotion. It has explicitly played down the distinction between cognitive and emotional mechanisms, noting the move toward affective-cognition in the wider cognitive sciences (Dukes et al., 2021). It has highlighted the key role of goals in meaning construction and has expanded on existing models by specifying the dynamic structure of appraisal. There is of course much to be said for how such a model is well suited to explore the non-prototypical and less stereotyped cases of emotional episodes. These may include misattribution (identifying the wrong antecedent object, such as performer instead of composer) or atypical appraisal (a fear of crowded spaces often sought by typical concert goers). However, the following hypotheses and implications are focused toward the typical.

### 4.3. Hypotheses & Implications

The CODA model proposes several hypotheses. These generally pertain to testing the value of goal-directed appraisal in emotional episodes induced by music. These hypotheses and their implications nonetheless deserve some explicit development with regards to their predictions and methodologies.

#### 4.3.1. Micro-Valences, Weighted Dimensions & Component Interactions

Hypotheses for appraisal's bidirectional interaction with core-affect comes form Kuppens et al. (2012). An experience sampling methodology (ESM) in real-life situations showed how appraisal and core-affect interact, focusing on the contribution of individual appraisals over appraisal patterns or interactions. The results indicated the valence-appraisal interaction was strongly marked by *motivational-consistency* (goal-conduciveness), as well as *coping-potential* (emotional and problem-focused) and *expectancy*. Supplementary to their hypotheses, it was also observed that an appraisal of *agency* showed small effects on valence ratings. The interaction between valence and appraisal was further conceptualized by more pleasant pre-exiting core-affect leading to more positive appraisal ratings. Arousal-appraisal interactions were characterized by *coping-potential* (problem-focused) and *agency*. The hypothesis for a *motivational relevance*—arousal interaction was not met. However, arousal influenced appraisals of *motivational relevance* and *expectancy*. The authors concluded that arousal plays a strong role in the pursuit of goals (p. 6). All together the evidence stresses a *dynamic interplay* (Kuppens et al., 2012) between components. No such appraisal based study yet exists in music to our knowledge. We hypothesize that the appraisals presented in our model will be relevant to music and its situational context. Moreover, appraisal dimensions will show bi-directional interactions with core-affect. Such a study would be straightforward to conduct as ESM is well developed in music research (e.g., Saarikallio et al., 2020b).

This interaction between appraisal and core-affect can be further conceptualized by the non-linear relationship between arousal and valence that more naturally represents a V-shape (Kuppens et al., 2017, Study 1). This is where more extreme ratings of valence (+ or -) naturally partner stronger subjective arousal ratings. An alternative hypothesis for the appraisal to arousal interaction noted by Kuppens et al. - one that highlights the micro-valenced nature of appraisal. Again such studies would be easy to generate through ESM, lab based, or online studies. Highly valenced (+ or -) music would show a V-shaped relationship with arousal. These effects may be amplified by the degree of goal-relevance a participant relates to the music and/or situation. Moreover, the V-shaped relationship between valence and arousal will show individual and cultural differences in the gradient of this V-shaped relationship.

One problem with Kuppens et al.'s (2012) study is that it is not clear whether individuals' ratings can be considered emotional episodes or moods. Moods in contrast being more diffuse, longer lived, and not directed at something (Russell, 2003). However, given that the same mechanisms in our model are hypothesized to underpin both cognitive and emotional cognition (i.e., degrees of more and less emotional cognition), this is not problematic for our interpretation. Instead, we suggest the evidence from Kuppens et al.'s study merely shows individual and contextual differences in more or less emotional cognition yet appraisals,are present in both conditions.

Appraisals also showed substantial correlations with each other (Kuppens et al., 2012). Though not the study's main focus, this suggests there are meaningful interactions between appraisal dimensions. We suggest, that changes in these interactions (the way appraisals constrain / amplify other appraisals) are to be seen as continuously changing weightings through the interaction between organism and environment. Extension to the proposed ESM experiments would benefit from more dynamic time-series analyses and Bayesian weightings. The studies proposed above can capture the importance and interaction of appraisal components as a snapshot in the music emotion process, although we have noted numerous times the process of ongoing meaning construction is a dynamic one. Such approaches have been developed for longitudinal ESM and diary studies (Asparouhov et al., 2018).

We hypothesize the appraisal interaction with core-affect and meta-experience will be characterized by micro-valences. What is unclear, is to what degree these micro-valences contribute to a macro-valence, or if this differs by individual, stimuli, situation or goal. Nonetheless, active testing that appraisals can contribute as micro-valences in the construction of meaning with music is another purposeful extension of the proposed experiment above and one that can be extended to more dynamic models.

Further evidence to inform hypotheses for micro-valences comes from Scherer et al. (2006). They looked at nine appraisal criteria and three-dimensional affective space (arousal, valence, potency) using the International Affective Picture System. They found the strongest valence correlation was with an *intrinsic pleasantness*/*goal-conduciveness* composite (0.99), as predicted. However, the observation of multiple correlations between appraisals and valence can be seen as supportive of micro-valences. Taken together with Kuppens et al. (2012) provides clear support for a micro-valenced interpretation of appraisal's interaction with core-affect. Beyond core-affect, a large-scale cross-cultural study (Soriano et al., 2013) using self-report measures of various components of emotion; including appraisal, action tendencies, bodily reactions, expressions, and feelings, similarly found strong support for the idea that appraisals are principally valenced structures (Fontaine et al., 2013) and that these valences load on to a “valence superfactor” (Scherer and Fontaine, 2013). Such a study in a musical context can be constructed using vignette methodologies. An methodological approach that has proved highly robust for appraisal (Robinson and Clore, 2001).

Micro-valences may also form part of inter-individual distinctions in emotional episodes through their effect on other components (e.g., why one person may find a piece of music more pleasant and energizing than another). Individuals with different goals (wishes, desires, beliefs) will appraise the same stimuli differently. Appraisals will therefore manifest themselves differently in the effect they have on other components, most prominently in valenced terms. Kuppens et al. (2012) notes individual differences in the relationship between appraisal and core affect. That is, people differed in the strength of effect appraisals had on core-affects. How different appraisals are weighted in different situations and by different individuals will form a key component in understanding contextual modifiers and would be observable through ESM or vignette methodologies (see The goal-directed hypothesis).

#### 4.3.2. The Goal-Directed Hypothesis

Why can the same piece of music in the same situation leave one person cold and another overcome with emotion? Our hypothesis is that the degree of goal-relevance / conduciveness influences the *intensity* of emotional episodes. That is, general cognitive processes must reach above an individually variable threshold of goal-relevance to become an emotional episode. The degree of goal-relevance will subsequently inform the intensity of that emotional episode. The change between less and more emotional cognition^7^ is therefore gradual (Moors et al., 2017). The intensity of an episode is therefore not fixed and can change over time. This can happen through greater weighting of a single goal, multiple simultaneous goals, or generation of new goals as an individual continues to engage more deeply with a stimulus. Furthermore, individual thresholds for goal-relevance are variable. That is, given different situations or prerequisite situations an individual's threshold for appraising something as goal-relevant changes. New stimuli may not be (subjectively) relevant enough to divert cognitive resources to it. We add that the degree of goal-relevance/conduciveness should show marked changes in autonomic reactions (sympathetic and para-sympathetic) and increase cognitive attention (orientation response) (Nieuwenhuis et al., 2011; Kreibig et al., 2012) beyond the relationship with psychoacoustic cues.

Individuals may prioritize different goals in the same situation as different expectations, thresholds, and predispositions for affordances exist (Moors, 2020). Musical behaviors like dancing are more common for some while attentive listening is for others (Saarikallio et al., 2020a). Emotional behaviors are thus guided by the utility of that behavior for achieving one's goals. For instance, dancing may enhance social bonding or physiological engagement for some, while others may not want to dance with the knowledge that a lack of coordination and timing (coping-potential) could distance them socially from their peers. Alternatively, a more pressing goal can take away the emotional engagement with a musical piece. For instance, the student who is anxious about their impending exams may not be able to enjoy their favorite music (mood-enhancement). Personal responses are a dynamic goal-driven process of meaning generation.

Interestingly, one could hypothetically also have a meta-appraisal of goal-relevance, in acknowledging a piece of music as perfect for an alternative goal or different situation. This meta-goal appraisal conceptualises one of the ways ongoing embodied and enacted learning allows for the development of new affordances. This notion ties in neatly with Huron's ITPRA model (Huron, 2006) which notes the relationship between imagination and appraisal. The interaction between affordances and imagination may prove an interesting and informative new direction for research linked closely to musical training and culture.

Such hypotheses require new goal-directed methodologies (Moors et al., 2017). This is experimentally equivalent to demonstrating the same piece of music for the same person can produce different behaviors based on different affordances. Such designs could be constructed around the functions music affords to different individuals or across different cultures (Schäfer et al., 2013) and be conducted through self-report methods. The identification of musical functions are a prerequisite to this (Gabrielsson, 2011; Boer et al., 2012; Maloney, 2019; Saarikallio et al., 2020a), including aesthetic (Zickfeld et al., 2019) and sonic (Schiavio et al., 2017a) affordances where music's value is explicitly tied into its function (Schäfer et al., 2012, 2013) and has shown systematic, albeit probabilistic, links with acoustic features (Mehr et al., 2019). What is key to these designs is to allow these elements to vary systematically.

A common criticism directed at appraisal theory (Scherer and Moors, 2019; and many music emotion studies Eerola and Vuoskoski, 2013) is the prevalence of self-report methodologies. Yet, such methodologies are still indicative of the underlying phenomena and allow for an informed baseline to emerge from which other approaches (biological, neural) can be compared (Eerola and Vuoskoski, 2013). Even forced-choice designs can be informative when well managed (Nelson and Russell, 2013). To expand the methodological arsenal further, implicit methodologies have been developed. Moors and De Houwer (2001) used a variation of the affective priming tasks to show how automatic appraisal of goal-conduciveness can influence an individual's responses. Similar results have been found using transcranial magnetic stimulation (Fischer et al., 2020). Goal-priming could be used in musical contexts. For instance, Céspedes-Guevara (in Warrenburg, 2020) shows how a composer's notes can influence audiences' interpretation of the music, implying an implicit goal to understand the composer's intended emotional communication, though many other goals could be constructed by individuals in a concert setting. Goals could be designed into a variety of musical scenarios, such as, hypothetical or real musical situations where typical musical functions are described. Similarly, showing differences between stimulus-driven outputs to goal-directed ones in approach / avoidance tasks would provide support for a goal-directed interpretation. Approach / avoidance musically could be considered several ways (e.g., listening time, purchasing cost etc.). The study of musical affordances over discrete emotions is paramount and applications may be particularly relevant when studying maladaptive listening behavior Carlson et al. (2015) where goal-directed approaches to behavior suggest changing the values of expectations, over changing the stimulus.

#### 4.3.3. Perceived vs. Induced Emotions

The distinction between perceived and induced emotions could be as simple as assessing a stimulus' goal-relevance. That is, a subjectively identified emotional episode emerges when a stimuli is seen as relevant enough to an individual to cross a goal-relevance threshold. A perceived assessment can emerge from an externally-directed goal (i.e., directed by the experimenter) to assess stimuli and pigeon-hole them into discrete categories. A participant can evaluate the available evidence: e.g.,in a music experiment—acoustic cues, changes in core-affect, physiological changes, appraisals, etc. However, devoid of much goal-relevance, evaluations remain relatively culturally standardised and the activity remains subjectively cognitively *cold*^8^.

#### 4.3.4. Utilitarian and Aesthetic Emotions

Within our approach, there is a desirable future where utilitarian and aesthetic emotions are also described within the same general components of cognition. One where aesthetic affordances, through a goal-directed mechanism, become the “to be studied phenomena.” This allows researchers to side-step arguments about which emotions are aesthetic or not and look toward behaviors and the interaction between components in the ongoing generation of meaning as the distinguishing features between aesthetic and utilitarian perspectives. Examples of the importance of goals have emerged in the aesthetic literature. Menninghaus et al. (2019) makes clear reference to the importance of goals and other appraisals in their conception of aesthetic emotions and notes their links with aesthetic functions and subjective feeling. However, Menninghaues et al. still continue to group their mechanisms around the organization of the emotion space (e.g., awe, interest, beauty). Meanwhile, we contend that the conversation must move beyond how the emotion space is organized.

Alternatively, we promote the study of aesthetic affordances through the reasons people have for engaging aesthetically. Again, we highlight the importance of moving beyond Kantian notions of disembodied and ‘disinterested’ (Huron, 2019) and readily acknowledge the incorporation of goals (Menninghaus et al., 2019; Zickfeld et al., 2019) in achieving this transition. However, we seek to adopt goal-directed action in its dynamic, embodied and enactive construction. Such goal-directed accounts may lead to distinctions in aesthetic affordances such as when attentive listening is afforded while removing the need for additional mechanisms.

#### 4.3.5. Recommendations

We finish with several recommendations for future research.

- As a priority future studies must place a direct focus on “nuisance variables” (familiarity, situation, individual differences) as systematically manipulated. These variables are not only understudied but directly pertain to music's relevance and meaning.
- Instead of exploring the full CODA model, individual parts of the model can be empirically tested (e.g., one or two appraisals) or interactions between just two components (e.g., goal-relevance and core-affect, physiological measures or attention).
- New methodologies with a focus on “nuisance variables” meaningfully moves the field beyond labeling emotions, and toward the emotional episodes. Such methodologies can be further extended to incorporate more implicit measures—EEG, TMS, reaction time, go no-go tasks.
- Extending methodologies, including self-report, to incorporate dynamic time-series analyses, allowing greater insight into the development of an emotional episode. Several examples of these types of analyses for different methodologies are noted through the paper as a template to guide future experiments.
- Develop new tools, allowing researchers to describe experiences differently, occurring at different levels and through different drivers. This approach advocates collecting descriptors of emotional responses that constitute these episodes, one where people can explain them in their own terms and giving reasons for them, rather than imposing structure of affects and mechanisms that dictate the choice of emotions and underlying drivers.
- Active testing of appraisals in relation to music requires careful review of the current literature, including aesthetics, and the development of new instruments. Such instruments should suit self-report formats and coding of free verbal/written reports. Current examples are limited by their focus upon grouping around discrete terms (Zentner et al., 2008).
- Research into appraisals should not be limited in scope. Embodied mechanisms such as entrainment, physiological responses, behaviors, and musical features can also be active features of appraisal.
- Direct comparisons with existing models in terms of coverage, realism, contribution to knowledge, and acknowledgment of the multiple influencing factors (structural, performance, listener, and context, Scherer and Zentner, 2001).
- Direct testing of goal-relevance as a mechanism to differentiate perceived and induced emotions.

## 5. Conclusion

This paper explored a skeptical perspective of current emotion science. Through studying key skeptical theories we accentuate the need to move away from “folk” terms and circular discussions about which emotion is present. Instead, we acknowledge the skeptical transition toward studying the emotion episode and its multidimensional components to better understand the underlying cognitive mechanisms. Furthermore, we examine goal-directed accounts of appraisal within the skeptical framework as directives for the relevance and meaning of a stimulus to an individual. We next examined two music-emotion models and investigated these models through the wider skeptical lens. We highlight how current theoretical constructs still seek to explain the underlying mechanisms through categorization. Furthermore, how the role of goal-directed appraisal, though uncontroversial in the wider affective sciences, remains to be meaningfully acknowledged within the construct of musical frameworks of emotion.

To address the gaps in music-emotion science we build on previous accounts (Eerola, 2017; Céspedes-Guevara and Eerola, 2018) and present the CODA model of musically induced emotional episodes. This model takes a skeptical approach and seeks to re-evaluate and recenter the role of goal-directed appraisal within music-emotion models. In doing so, we have developed a multidimensional appraisal framework that, through goal-directed processes, links our model to embodied and enactive schools of thought and is closely tied to the concept of musical affordance (sonic and situational). Importantly, we content that this model places emotional episodes induced by music explicitly within the wider framework of general cognitive/affective mechanism which allows for music-emotion science to be examined more universally as situated within the study of the human experience.

Finally, we offer several hypotheses derived from the CODA model. These hypotheses address current theoretical issues such as distinctions between aesthetic and utilitarian emotions, perceived and induced emotions, as well as individual and situational differences in emotional intensity and valence. To test our hypotheses, we develop existing and new methodologies before offering a list of critical recommendations for future research. Altogether, we show that the future of music and emotion science must reconsider the prevalence and impact of an individual's motivations in the generation of musical emotional episodes – a question that can only be addressed by looking at an individual's goals. Simply, we insist that the important questions are no longer *why this emotion?*, but *why this music, in this moment, for this individual?*

## Data Availability Statement

The original contributions presented in the study are included in the article/Supplementary Material, further inquiries can be directed to the corresponding author.

## Author Contributions

TL synthesized the literature, developed the key aspects of the CODA model, wrote the dominant part of the text, and contributed to visualizations. TE contributed to background theories, reshaped the CODA model, the visualizations, the overall structure of the argument, and contributed to writing the text. Both authors contributed to the article and approved the submitted version.

## Funding

This research was funded by an Arts and Humanities Research Council scholarship (grant code: AH/L503927/1) awarded to TL through the Northern Bridge Doctoral Training Partnership. Open access publication fees were contributed by Durham University's Open Access team.

## Conflict of Interest

The authors declare that the research was conducted in the absence of any commercial or financial relationships that could be construed as a potential conflict of interest.

## Publisher's Note

All claims expressed in this article are solely those of the authors and do not necessarily represent those of their affiliated organizations, or those of the publisher, the editors and the reviewers. Any product that may be evaluated in this article, or claim that may be made by its manufacturer, is not guaranteed or endorsed by the publisher.

## Footnotes

^1^This term is also often referred to as reappraisal though this concept is less well defined and can be associated with both D-A (Dimensional-Appraisal) and discrete-appraisal.

^2^This term is also often referred to as reappraisal though this concept is less well defined and can be associated with both D-A and discrete-appraisal.

^3^Goals defined here broadly, as learnt as well as innate and encompassing the desires, motivations, wishes, and needs of an individual (Moors, 2020).

^4^Dimensional in italics here to highlight the conceptualization of appraisals as dimensional by all skeptical theories but avoid explicit reference to D-A theory as more important than other skeptical theories in our interpretation of a skeptical model of musical emotions.

^5^Separate from core-affect but represented in the same two-dimensions. Affective quality is a cold cognitive assessment of a stimulus with regards to its ability to cause changes in core-affect. It is therefore located within the stimulus not the person (Russell, 2003). It is made through evaluations, attitudes, liking/disliking and is often combined with other information processing constructs.

^6^Russell ties the concept closely with evaluations, attitudes, and liking. The distinction lies in Russell's contention that affective quality is a cognitively cold assessment that can be represented in the same two dimensions as core-affect. Our model implies multi-dimensionality (instead of the multivariate bi-polarity present by Russell, 2017b) which can shift between hot and cold cognition while keeping the explanatory capacity of one-dimensional valence.

^7^Often referred to as a distinction between cold and hot cognition to try and separate cognitive and emotional mechanisms. This is a distinction we do not seek to endorse.

^8^Cognitively *cold* here used only to refer to the subjective experience. It is not to imply a meaningful distinction between cognitive and emotional mechanisms.

## References

[B1] Arnold M. B. (1960). Emotion and Personality, Vol. 1, Psychological Aspects. New York, NY: Columbia University Press.

[B2] Asparouhov T. Hamaker E. L. Muthén B. (2018). Dynamic structural equation models. Struct. Equat. Model. Multidisc. J. 25, 359–388. 10.1080/10705511.2017.1406803.

[B3] Aue T. Scherer K. R. (2008). Appraisal-driven somatovisceral response patterning: effects of intrinsic pleasantness and goal conduciveness. Biol. Psychol. 79, 158–164. 10.1016/j.biopsycho.2008.04.004 18495321

[B4] Aue T. Scherer K. R. (2011). Effects of intrinsic pleasantness and goal conduciveness appraisals on somatovisceral responding: somewhat similar, but not identical. Biol. Psychol. 86, 65–73. 10.1016/j.biopsycho.2010.10.008 21029762

[B5] Australia A. N. (2019, Jun 14). Hawke memorial: William Barton and the Sydney Symphony Orchestra play Down Under | ABC news [video]. YouTube.

[B6] Balleine B. Dickinson A. (1998). Consciousness—the interface between affect and cognition. in Consciousness and Human Identity, ed Cornwell J. (Oxford: Oxford University Press), 57–85.

[B7] Barradas G. (2017). A cross-cultural approach to psychological mechanisms underlying emotional reactions to music.

[B8] Barrett L. F. (2006). Solving the emotion paradox: categorization and the experience of emotion. Pers. Soc. Psychol. Rev. 10, 20–46. 10.1207/s15327957pspr1001_2 16430327

[B9] Barrett L. F. (2017). How Emotions Are Made: The Secret Life of the Brain. London: Pan Books.

[B10] Barrett L. F. Satpute A. B. (2013). Large-scale brain networks in affective and social neuroscience: towards an integrative functional architecture of the brain. Curr. Opin. Neurobiol. 23, 361–372. 10.1016/j.conb.2012.12.012 23352202 PMC4119963

[B11] Bechtel W. Shagrir O. (2015). The non-redundant contributions of marr's three levels of analysis for explaining information-processing mechanisms. Top. Cogn. Sci. 7, 312–322. 10.1111/tops.12141 25900887

[B12] Belyk M. Brown S. (2014). The acoustic correlates of valence depend on emotion family. J. Voice 28, 523.e9–523.e18. 10.1016/j.jvoice.2013.12.007 24495430

[B13] Berlyne D. E. (1971). Aesthetics and Psychobiology. New York, NY: Appleton-Century-Crofts.

[B14] Berridge K. C. Kringelbach M. L. (2013). Neuroscience of affect: brain mechanisms of pleasure and displeasure. Curr. Opin. Neurobiol. 23, 294–303. 10.1016/j.conb.2013.01.017 23375169 PMC3644539

[B15] Boer D. Fischer R. Tekman H. G. Abubakar A. Njenga J. Zenger M. (2012). Young people's topography of musical functions: personal, social and cultural experiences with music across genders and six societies. Int. J. Psychol. 47, 355–369. 10.1080/00207594.2012.656128 22506759

[B16] Brosch T. Sander D. (2013). Comment: the appraising brain: towards a neuro-cognitive model of appraisal processes in emotion. Emotion Rev. 5, 163–168. 10.1177/1754073912468298

[B17] Brosch T. Scherer K. R. Grandjean D. M. Sander D. (2013). The impact of emotion on perception, attention, memory, and decision-making. Swiss Med. Wkly 143, w13786. 10.4414/smw.2013.13786 23740562

[B18] Brunswik E. (1956). Perception and the Representative Design of Psychological Experiments. Berkeley; Los Angeles, CA: University of California Press.

[B19] Cabanac M. (2010). The dialectics of pleasure, in Pleasures of the Brain, eds Kringelbach M. L. Berridge K. C. (Oxford, UK: Oxford University Press), 113–124.

[B20] Cacioppo J. T. Berntson G. G. Larsen J. T. Poehlmann K. M. Ito T. A. others (2000). The psychophysiology of emotion, in Handbook of Emotions, eds Lewis M. Haviland-Jones J. M. (New York, NY: Guilford Press), 173–191.

[B21] Cacioppo J. T. Gardner W. L. Berntson G. G. (1999). The affect system has parallel and integrative processing components: form follows function. J. Pers. Soc. Psychol. 76, 839–855.

[B22] Carlson E. Saarikallio S. Toiviainen P. Bogert B. Kliuchko M. Brattico E. (2015). Maladaptive and adaptive emotion regulation through music: a behavioral and neuroimaging study of males and females. Front. Hum. Neurosci. 9, 466. 10.3389/fnhum.2015.00466 26379529 PMC4549560

[B23] Céspedes-Guevara J. Eerola T. (2018). Music communicates affects, not basic emotions–a constructionist account of attribution of emotional meanings to music. Front. Psychol. 9, 215. 10.3389/fpsyg.2018.00215 29541041 PMC5836201

[B24] Céspedes-Guevara Julian A. (2021). A constructionist theory of music induced emotions. PsyArXiv. Available at: psyarxiv.com/sfzm2.

[B25] Clarke E. F. (2005). Ways of Listening: An Ecological Approach to the Perception of Musical Meaning. Oxford: Oxford University Press.

[B26] Clark-Polner E. Wager T. Satpute A. Barrett L. (2016). Neural fingerprinting: meta-analysis, variation, and the search for brain-based essences in the science of emotion, in The handbook of emotion, eds Barrett L. F. Lewis M. Haviland-Jones J. M. (London: Guilford Press) 146–165.

[B27] Clayton M. (2017). The Ethnography of Embodied Music Interaction. London: Routledge.

[B28] Clore G. L. Ortony A. (2000). Cognition in emotion: always, sometimes, or never, in Cognitive Neuroscience of Emotion, eds Lane R. D. Nadel L. (Oxford: Oxford University Press), 24–61.

[B29] Colombetti G. (2014). The Feeling Body: Affective Science Meets the Enactive Mind. Cambridge, MA: MIT Press.

[B30] Colombetti G. (2018). The oxford handbook of 4E cognition. in The oxford Handbook of 4E Cognition, eds. Albert Newen Leon De Bruin Gallagher S. (Oxford: Oxford University Press), 571–588. 10.1093/oxfordhb/9780198735410.013.31

[B31] Coutinho E. Scherer K. R. (2017). Introducing the geneva music-induced affect checklist (GEMIAC): a brief instrument for the rapid assessment of musically induced emotions. Music Percept. 34, 371–386. 10.1525/mp.2017.34.4.371

[B32] Crivelli C. Jarillo S. Russell J. A. Fernández-Dols J.-M. (2016). Reading emotions from faces in two indigenous societies. J. Exp. Psychol. 145, 830–843. 10.1037/xge0000172 27100308

[B33] Dukes D. Abrams K. Adolphs R. Ahmed M. E. Beatty A. Berridge K. C.. (2021). The rise of affectivism. Nat. Hum. Behav. 5, 816–820. 10.1038/s41562-021-01130-8 34112980 PMC8319089

[B34] Eder A. B. Hommel B. (2013). Anticipatory control of approach and avoidance: An ideomotor approach. Emotion Rev. 5, 275–279. 10.1177/1754073913477505

[B35] Eerola T. (2011). Are the emotions expressed in music genre-specific? An audio-based evaluation of datasets spanning classical, film, pop and mixed genres. J. New Music Res. 40, 349–366. 10.1080/09298215.2011.602195

[B36] Eerola T. (2017). Springer handbook of systematic musicology, in Springer Handbook of Systematic Musicology, ed Bader R. (Berlin; Heidelberg: Springer), 539–554.

[B37] Eerola T. Peltola H.-R. (2016). Memorable experiences with sad music-reasons, reactions and mechanisms of three types of experiences. PLoS ONE 11, e0157444. 10.1371/journal.pone.0157444 27300268 PMC4907454

[B38] Eerola T. Vuoskoski J. K. (2013). A review of music and emotion studies: approaches, emotion models, and stimuli. Music Percept 30, 307–340. 10.1525/mp.2012.30.3.307

[B39] Egermann H. Reuben F. (2020). “Beauty is how you feel inside”: aesthetic judgments are related to emotional responses to contemporary music. Front. Psychol. 11, 510029. 10.3389/fpsyg.2020.510029 33281651 PMC7691637

[B40] Ekman P. Levenson R. W. Friesen W. V. (1983). Autonomic nervous system activity distinguishes among emotions. Science 221, 1208–1210. 6612338 10.1126/science.6612338

[B41] Ellsworth P. C. (2013). Appraisal theory: old and new questions. Emotion Rev. 5, 125–131. 10.1177/175407391246361730038591

[B42] Ellsworth P. C. Scherer K. R. (2003). Handbook of Affective Sciences. Oxford, UK: Oxford University Press.

[B43] Fischer M. Fini C. Brass M. Moors A. (2020). Early approach and avoidance tendencies can be goal-directed: support from a transcranial magnetic stimulation study. Cogn. Affect. Behav. Neurosci. 20, 648–657. 10.3758/s13415-020-00793-6 32333239

[B44] Flaig N. Large E. W. (2014). Dynamic musical communication of core affect. Front. Psychol. 5, 72. 10.3389/fpsyg.2014.00072 24672492 PMC3956121

[B45] Fontaine J. R. Scherer K. R. Soriano C. (2013). Components of Emotional Meaning: A Sourcebook. Oxford: Oxford University Press.

[B46] Frijda N. H. (2009). Emotion experience and its varieties. Emotion Revi. 1, 264–271. 10.1177/1754073909103595

[B47] Frijda N. H. (2017). The Laws of Emotion. New York, NY: Psychology Press.

[B48] Friston K. J. Mechelli A. Turner R. Price C. J. (2000). Nonlinear responses in fMRI: the balloon model, volterra kernels, and other hemodynamics. Neuroimage 12, 466–477. 10.1006/nimg.2000.0630 10988040

[B49] Gabrielsson A. (2011). Strong Experiences With Music: Music is Much More Than Just Music. Oxford: Oxford University Press.

[B50] Gabrielsson A. Lindström E. (2010). The role of structure in the musical expression of emotions, in Handbook of Music and Emotion: Theory, Research, Applications, ed Juslin P. N. Sloboda J. A. (Oxford, UK: Oxford University Press), 367–400.

[B51] Gibson J. J. (1977). The Theory of Affordances, in The People, Place, and Space Reader, eds Gieseking J. J. Mangold W. Katz C. Low S. Saegert S. (Hilldale, PA: Routledge).

[B52] Gigerenzer G. (2021). Embodied heuristics. Front. Psychol. 12, 4243. 10.3389/fpsyg.2021.711289 34858251 PMC8631174

[B53] Gigerenzer G. Gaissmaier W. (2011). Heuristic decision making. Ann. Rev. Psychol. 62, 451–482. 10.1146/annurev-psych-120709-145346 21126183

[B54] Haidt J. Keltner D. (1999). Culture and facial expression: Open-ended methods find more expressions and a gradient of recognition. Cogn. Emotion 13, 225–266.

[B55] Hallam S. Cross I. Thaut M. (2016). Oxford Handbook of Music Psychology, 2nd Edn. Oxford: Oxford University Press.

[B56] Herbert R. Dibben N. (2018). Making sense of music: meanings 10-to 18-year-olds attach to experimenter-selected musical materials. Psychol. Music 46, 375–391. 10.1177/0305735617713118

[B57] Holochwost S. J. Izard C. E. (2008). Evidence from young children regarding emotional responses to music. Behav. Brain Sci. 31, 581–582. 10.1017/S0140525X08005360

[B58] Hu X. Wang F. Zhang D. (2022). Similar brains blend emotion in similar ways: Neural representations of individual difference in emotion profiles. Neuroimage 247, 118819. 10.1016/j.neuroimage.2021.118819 34920085

[B59] Hunter P. G. Schellenberg E. G. Schimmack U. (2008). Mixed affective responses to music with conflicting cues. Cogn. Emotion 22, 327–352. 10.1080/02699930701438145

[B60] Hunter P. G. Schellenberg E. G. Schimmack U. (2010). Feelings and perceptions of happiness and sadness induced by music: similarities, differences, and mixed emotions. Psychol. Aesth. Creat. Arts 4, 47. 10.1037/a0016873

[B61] Huron D. (2006). Sweet Anticipation: Music and the Psychology of Expectation. London: MIT Press.

[B62] Huron D. (2015). Cues and signals: an ethological approach to music-related emotion. Signata Annales des sémiotiques/Annals of Semiotics. 6, 331–351. 10.4000/signata.1115

[B63] Huron D. (2016). Music and meaning, in The Oxford Handbook of Music Psychology, eds Hallam S. Cross I. Thaut M. (Oxford: Oxford University Press), 233–245.

[B64] Huron D. (2019). Musical aesthetics: uncertainty and surprise enhance our enjoyment of music. Curr. Biol. 29, R1238–R1240. 31794756 10.1016/j.cub.2019.10.021

[B65] Inzlicht M. Bartholow B. D. Hirsh J. B. (2015). Emotional foundations of cognitive control. Trends Cogn. Sci. 19, 126–132. 10.1016/j.tics.2015.01.004 25659515 PMC4348332

[B66] Ito T. Cacioppo J. (2005). Variations on a human universal: individual differences in positivity offset and negativity bias. Cogn. Emotion 19, 1–26. 10.1080/02699930441000120

[B67] James W. (1884). What is an emotion? Mind 9, 188–205.

[B68] Juslin P. N. (2013). From everyday emotions to aesthetic emotions: Towards a unified theory of musical emotions. Phys. Life Rev. 10, 235–266. 10.1016/j.plrev.2013.05.008 23769678

[B69] Juslin P. N. (2019). Musical Emotions Explained: Unlocking the Secrets of Musical Affect. Oxford: Oxford University Press.

[B70] Juslin P. N. Barradas G. Eerola T. (2015). From sound to significance: exploring the mechanisms underlying emotional reactions to music. Am. J. Psychol. 128, 281–304. 10.5406/amerjpsyc.128.3.0281 26442337

[B71] Juslin P. N. Harmat L. Eerola T. (2014). What makes music emotionally significant? Exploring the underlying mechanisms. Psychol. Music 42, 599–623. 10.1177/0305735613484548

[B72] Juslin P. N. Laukka P. (2003). Communication of emotions in vocal expression and music performance: different channels, same code? Psychol. Bull. 129, 770–814. 10.1037/0033-2909.129.5.770 12956543

[B73] Juslin P. N. Liljeström S. Laukka P. Västfjäll D. Lundqvist L.-O. (2011). Emotional reactions to music in a nationally representative sample of swedish adults: prevalence and causal influences. Musicae Scientiae 15, 174–207. 10.1177/1029864911401169

[B74] Juslin P. N. Liljeström S. Vas¨tfjall D. Barradas G. Silva A. (2008). An experience sampling study of emotional reactions to music: Listener, music, and situation. Emotion 8, 668–683. 18837617 10.1037/a0013505

[B75] Juslin P. N. Sloboda J. (2010). Handbook of Music and Emotion: Theory, Research, Applications. Oxford: Oxford University Press.

[B76] Juslin P. N. V"astfj"all D. (2008). Emotional responses to music: The need to consider underlying mechanisms. Behav. Brain Sci. 31, 559–575. 10.1017/S0140525X08005293 18826699

[B77] Kafkas A. Montaldi D. (2014). Two separate, but interacting, neural systems for familiarity and novelty detection: a dual-route mechanism. Hippocampus 24, 516–527. 10.1002/hipo.22241 24436072

[B78] Kant I. (2001). Critique of the Power of Judgment, eds Guyer P. Matthews T. (Cambridge: Cambridge University Press).

[B79] Kivy P. (1981). The Corded Shell: Reflections on Musical Expression. Princeton, NJ: Princeton University Press.

[B80] Kivy P. (1989). Sound Sentiment: An Essay on the Musical Emotions, Including the Complete Text of the Corded Shell. Philadelphia, PA: Temple University Press.

[B81] Koelsch S. Jacobs A. M. Menninghaus W. Liebal K. Klann-Delius G. Von Scheve C.. (2015). The quartet theory of human emotions: an integrative and neurofunctional model. Phys. Life Rev. 13, 1–27. 10.1016/j.plrev.2015.03.001 25891321

[B82] Konečni V. J. (2008). A skeptical position on “musical emotions” and an alternative proposal. Behav. Brain Sci. 31, 582–584. 10.1017/S0140525X08005372

[B83] Kreibig S. D. Gendolla G. H. Scherer K. R. (2012). Goal relevance and goal conduciveness appraisals lead to differential autonomic reactivity in emotional responding to performance feedback. Biol. Psychol. 91, 365–375. 10.1016/j.biopsycho.2012.08.007 22947258

[B84] Kringelbach M. L. Berridge K. C. (2015). The psychological construction of emotion, in eds Barrett L. F. Russell J. A. (New York, NY: Guilford Press), 229–248.

[B85] Kuppens P. Champagne D. Tuerlinckx F. (2012). The dynamic interplay between appraisal and core affect in daily life. Front. Psychol. 3, 380 10.3389/fpsyg.2012.00380 23060842 PMC3466066

[B86] Kuppens P. Tuerlinckx F. Yik M. Koval P. Coosemans J. Zeng K. J.. (2017). The relation between valence and arousal in subjective experience varies with personality and culture. J. Pers. 85, 530–542. 27102867 10.1111/jopy.12258

[B87] Laukka P. Elfenbein H. A. (2012). Emotion appraisal dimensions can be inferred from vocal expressions. Soc. Psychol. Pers. Sci. 3, 529–536. 10.1177/1948550611428011 29291085 PMC5717659

[B88] LeDoux J. E. (2008). Emotional colouration of consciousness: How feelings come about, in Frontiers of consciousness: Chichele lectures (Oxford: Oxford University Press), 69–130.

[B89] Lerner J. S. Keltner D. (2001). Fear, anger, and risk. J. Pers. Soc. Psychol. 81, 146. 10.1037/0022-3514.81.1.146 11474720

[B90] Lewis M. D. (2005). Bridging emotion theory and neurobiology through dynamic systems modeling. Behav. Brain Sci. 28, 169–194. 10.1017/s0140525x0500004x 16201458

[B91] Madison G. (2008). What about the music? Music-specific functions must be considered in order to explain reactions to music. Behav. Brain Sci. 31, 587–587. 10.1017/S0140525X08005414

[B92] Maksimainen J. Eerola T. Saarikallio S. (2019). Ambivalent emotional experiences of everyday visual and musical objects. Sage Open 9, 2158244019876319. 10.1177/2158244019876319

[B93] Malmgren H. (2008). Identifying and individuating the psychological mechanisms that underlie musical emotions. Behavioral and Brain Sciences 31, 587–588.

[B94] Maloney L. (2019). Music Like Water: Exploring the Functions of Music Through Thematic Bibliometric Analysis and Comparative ESM Study. (Doctoral dissertation), University of York. Available online at: https://ethos.bl.uk/OrderDetails.do?uin=uk.bl.ethos.822347.

[B95] Marr D. (1982). Vision: A Computational Investigation Into the Human Representation and Processing of Visual Information. New York, NY: W. H. Freeman; Company.

[B96] Mehr S. A. Singh M. Knox D. Ketter D. M. Pickens-Jones D. Atwood S.. (2019). Universality and diversity in human song. Science 366, eaax0868. 10.1126/science.aax0868 31753969 PMC7001657

[B97] Menninghaus W. Wagner V. Wassiliwizky E. Schindler I. Hanich J. Jacobsen T.. (2019). What are aesthetic emotions? Psychol. Rev. 126, 171–195. 10.1037/rev0000135 30802122

[B98] Merriam A. P. Merriam V. (1964). The Anthropology of Music. Evanston, IL: Northwestern University Press.

[B99] Meyer L. B. (1956). Emotion and Meaning in Music. Chicago, IL: University of Chicago Press.

[B100] Moors A. (2013). On the causal role of appraisal in emotion. Emotion Rev. 5, 132–140. 10.1177/1754073912463601

[B101] Moors A. (2014). Flavors of appraisal theories of emotion. Emotion Rev. 6, 303–307. 10.1177/1754073914534477

[B102] Moors A. (2017). Integration of two skeptical emotion theories: Dimensional appraisal theory and Russell's psychological construction theory. Psychol. Inquiry 28, 1–19. 10.1080/1047840X.2017.1235900

[B103] Moors A. (2020). Appraisal theory of emotion, in Encyclopedia of Personality and Individual Differences, eds Zeigler-Hill V. Shackelford T. K. (Cham: Springer International Publishing), 232–240.

[B104] Moors A. Boddez Y. De Houwer J. (2017). The power of goal-directed processes in the causation of emotional and other actions. Emotion Rev. 9, 310–318. 10.1177/1754073916669595

[B105] Moors A. De Houwer J. (2001). Automatic appraisal of motivational valence: motivational affective priming and simon effects. Cogn. Emotion 15, 749–766. 10.1080/02699930143000293

[B106] Moors A. Ellsworth P. C. Scherer K. R. Frijda N. H. (2013). Appraisal theories of emotion: State of the art and future development. Emotion Rev. 5, 119–124. 10.1177/1754073912468165

[B107] Moors A. Fischer M. (2019). Demystifying the role of emotion in behaviour: Toward a goal-directed account. Cogn. Emotion 33, 94–100. 10.1080/02699931.2018.1510381 30102113

[B108] Moors A. Kuppens P. (2008). Distinguishing between two types of musical emotions and reconsidering the role of appraisal. Behav. Brain Sci. 31, 588–589. 10.1017/S0140525X08005438

[B109] Moors A. Scherer K. R. (2013). Handbook of cognition and emotion, in Handbook of Cognition and Emotion, eds Robinson M. D. Watkins E. R. Harmon-Jones E. (New York, NY: Guilford Press), 135–155.

[B110] Nelson N. L. Russell J. A. (2013). Universality revisited. Emotion Rev. 5, 8–15. 10.1177/1754073912457227

[B111] Newen A. De Bruin L. Gallagher S. (2018). The Oxford Handbook of 4E Cognition. Oxford: Oxford University Press.

[B112] Nieuwenhuis S. De Geus E. J. Aston-Jones G. (2011). The anatomical and functional relationship between the P3 and autonomic components of the orienting response. Psychophysiology 48, 162–175. 10.1111/j.1469-8986.2010.01057.x 20557480 PMC3797154

[B113] Nordström H. Laukka P. Thingujam N. S. Schubert E. Elfenbein H. A. (2017). Emotion appraisal dimensions inferred from vocal expressions are consistent across cultures: a comparison between australia and India. R. Soc. Open Sci. 4, 170912. 10.1098/rsos.170912 29291085 PMC5717659

[B114] Oatley K. Johnson-Laird P. N. (1987). Towards a cognitive theory of emotions. Cogn. Emotion 1, 29–50.

[B115] Park M. Thom J. Mennicken S. Cramer H. Macy M. (2019). Global music streaming data reveal diurnal and seasonal patterns of affective preference. Nat. Hum. Behav. 3, 230–236. 10.1038/s41562-018-0508-z 30953008

[B116] Parkinson B. (1997). Untangling the appraisal-emotion connection. Pers. Soc. Psychol. Rev. 1, 62–79. 15647129 10.1207/s15327957pspr0101_5

[B117] Pfaff D. W. (2006). Brain Arousal and Information Theory: Neural and Genetic Mechanisms. Cambridge, MA: Harvard University Press.

[B118] Posner J. Russell J. A. Peterson B. S. (2005). The circumplex model of affect: an integrative approach to affective neuroscience, cognitive development, and psychopathology. Dev. Psychopathol. 17, 715–734. 10.1017/S0954579405050340 16262989 PMC2367156

[B119] Quigley K. S. Barrett L. F. (2014). Is there consistency and specificity of autonomic changes during emotional episodes? Guidance from the conceptual act theory and psychophysiology. Biol. Psychol. 98, 82–94. 10.1016/j.biopsycho.2013.12.013 24388802 PMC4041070

[B120] Randall W. M. Rickard N. S. (2017). Reasons for personal music listening: a mobile experience sampling study of emotional outcomes. Psychol. Music 45, 479–495. 10.1177/0305735616666939

[B121] Reybrouck M. Eerola T. (2017). Music and its inductive power: a psychobiological and evolutionary approach to musical emotions. Front. Psychol. 8, 494. 10.3389/fpsyg.2017.00494 28421015 PMC5378764

[B122] Robinson J. (2005). Deeper than reason: Emotion and its role in literature, music, and art. Oxford University Press on Demand.

[B123] Robinson J. (2008). Do all musical emotions have the music itself as their intentional object? Behav. Brain Sci. 31, 592–593. 10.1017/S0140525X08005475

[B124] Robinson M. D. Clore G. L. (2001). Simulation, scenarios, and emotional appraisal: testing the convergence of real and imagined reactions to emotional stimuli. Pers. Soc. Psychol. Bull. 27, 1520–1532. 10.1177/01461672012711012

[B125] Roseman I. J. (2017). Transformative events: Appraisal bases of passion and mixed emotions. Emotion Rev. 9, 133–139. 10.1177/1754073916661764

[B126] Russell J. A. (2003). Core affect and the psychological construction of emotion. Psychol. Rev. 110, 145–172. 10.1037/0033-295x.110.1.145 12529060

[B127] Russell J. A. (2009). Emotion, core affect, and psychological construction. Cogn. Emotion 23, 1259–1283. 10.1080/0269993090280937512529060

[B128] Russell J. A. (2012). From a psychological constructionist perspective, in Categorical Versus Dimensional Models of Affect: A Seminar on the Theories of Panksepp and Russell, eds Zachar P. Ellis R. D. (Amsterdam: John Benjamins Publishing), 79–118.

[B129] Russell J. A. (2017a). Emotion recognition: Is it universal? Available online at: http://dspace.library.uvic.ca/handle/1828/8076

[B130] Russell J. A. (2017b). Mixed emotions viewed from the psychological constructionist perspective. Emotion Rev. 9, 111–117. 10.1177/175407391663965830602318

[B131] Saarikallio S. (2011). Music as emotional self-regulation throughout adulthood. Psychol. Music 39, 307–327. 10.1177/0305735610374894

[B132] Saarikallio S. Alluri V. Maksimainen J. Toiviainen P. (2020a). Emotions of music listening in Finland and in India: comparison of an individualistic and a collectivistic culture. Psychol. Music. 49, 989–1005. 10.1177/0305735620917730

[B133] Saarikallio S. H. Randall W. M. Baltazar M. (2020b). Music listening for supporting adolescents' sense of agency in daily life. Front. Psychol. 10, 2911. 10.3389/fpsyg.2019.02911 32010014 PMC6960221

[B134] Schäfer T. Sedlmeier P. Städtler C. Huron D. (2013). The psychological functions of music listening. Front. Psychol. 4, 511. 10.3389/fpsyg.2013.00511 23964257 PMC3741536

[B135] Schäfer T. Tipandjan A. Sedlmeier P. (2012). The functions of music and their relationship to music preference in India and Germany. Int. J. Psychol. 47, 370–380. 10.1080/00207594.2012.688133 22721000

[B136] Scherer K. Zentner M. (2008). Music evoked emotions are different–more often aesthetic than utilitarian. Behav. Brain Sci. 31, 595–596. 10.1017/S0140525X08005505

[B137] Scherer K. R. (1986). Vocal affect expression: a review and a model for future research. Psychol. Bull. 99, 143–165. 10.1037/0033-2909.99.2.143 3515381

[B138] Scherer K. R. (2000). Psychological models of emotion, in The Neuropsychology of Emotion, ed Borod J. C. (Oxford: Oxford University Press), 137–162.

[B139] Scherer K. R. (2009a). Emotions are emergent processes: They require a dynamic computational architecture. Philos. Trans. R. Soc. B Biol. Sci. 364, 3459–3474. 10.1098/rstb.2009.0141 19884141 PMC2781886

[B140] Scherer K. R. (2009b). The dynamic architecture of emotion: evidence for the component process model. Cogn. Emotion 23, 1307–1351. 10.1080/02699930902928969

[B141] Scherer K. R. Brosch T. (2009). Culture-specific appraisal biases contribute to emotion dispositions. Eur. J. Pers. 23, 265–288. 10.1002/per.714

[B142] Scherer K. R. Coutinho E. (2013). How music creates emotion: A multifactorial process approach, in The Emotional Power of Music: Multidisciplinary Perspectives on Musical Arousal, Expression, and Social Control, eds Cochrane F. B. Scherer T K. R. (Oxford, UK: Oxford University Press), 121–145.

[B143] Scherer K. R. Dan E. Flykt A. (2006). What determines a feeling's position in affective space? A case for appraisal. Cogn. Emotion 20, 92–113. 10.1080/02699930500305016

[B144] Scherer K. R. Fontaine J. (2013). Driving the emotion process: The appraisal component, in Components of Emotional Meaning: A Sourcebook, eds Fontaine J. Scherer K. R. Soriano C. (Oxford: Oxford University Press), 186–209.

[B145] Scherer K. R. Moors A. (2019). The emotion process: Event appraisal and component differentiation. Ann. Rev. Psychol. 70, 719–745. 10.1146/annurev-psych-122216-011854 30110576

[B146] Scherer K. R. Mortillaro M. Mehu M. (2017). The science of facial expression, in The Science of Facial Expression, eds Dols J. M. F. Russell J. A. (Oxford: Oxford University Press), 353–373.

[B147] Scherer K. R. Zentner M. R. (2001). Emotional effects of music: Production rules, in Music Emotion: Theory and Research eds Juslin P. N. Sloboda J. A. (Oxford: Oxford University Press), 361, 392.

[B148] Schiavio A. Schyff D. van der Cespedes-Guevara J. Reybrouck M. (2017a). Enacting musical emotions. Sense-making, dynamic systems, and the embodied mind. Phenomenol. Cogn. Sci. 16, 785–809. 10.1007/s11097-016-9477-8

[B149] Schiavio A. Schyff D. van der Kruse-Weber S. Timmers R. (2017b). When the sound becomes the goal. 4E cognition and teleomusicality in early infancy. Front. Psychol. 8, 1585. 10.3389/fpsyg.2017.01585 28993745 PMC5622185

[B150] Schindler I. Hosoya G. Menninghaus W. Beermann U. Wagner V. Eid M.. (2017). Measuring aesthetic emotions: a review of the literature and a new assessment tool. PLoS ONE 12, e0178899. 10.1371/journal.pone.0178899 28582467 PMC5459466

[B151] Scruton R. (1999). The Aesthetics of Music. Oxford: Oxford University Press.

[B152] Shuman V. Sander D. Scherer K. R. (2013). Levels of valence. Front. Psychol. 4, 261. 10.3389/fpsyg.2013.00261 23717292 PMC3651968

[B153] Siegel E. H. Sands M. K. Van den Noortgate W. Condon P. Chang Y. Dy J.. (2018). Emotion fingerprints or emotion populations? A meta-analytic investigation of autonomic features of emotion categories. Psychol. Bull. 144, 343–393. 10.1037/bul0000128 29389177 PMC5876074

[B154] Silvia P. J. (2005a). Cognitive appraisals and interest in visual art: Exploring an appraisal theory of aesthetic emotions. Empirical Stud. Arts 23, 119–133. 10.2190/12AV-AH2P-MCEH-289E

[B155] Silvia P. J. (2005b). What is interesting? Exploring the appraisal structure of interest. Emotion 5, 89–102. 10.1037/1528-3542.5.1.89 15755222

[B156] Silvia P. J. (2006). Artistic training and interest in visual art: Applying the appraisal model of aesthetic emotions. Empirical studies of the arts 24, 139–161.

[B157] Sloboda J. A. Juslin P. N. (2010). At the interface between the inner and outer world, in Handbook of Music and Emotion (Oxford: Oxford University Press), 73–97.

[B158] Smith C. A. Lazarus R. S. (1991). Emotion and adaptation, in Handbook of Personality: Theory and Research, ed Pervin L. A. (New York, NY: Guilford Press), 609–637.

[B159] Soriano C. Fontaine J. Scherer K. R. Akçalan Akirmak G. Alarcçn P. Alonso-Arbiol I.. (2013). Cross-cultural data collection with the GRID instrument, in Components of Emotional Meaning: A Sourcebook, eds Fontaine J. R. Scherer K. R. Soriano C. (Oxford: Oxford University Press), 98–105.

[B160] Thompson W. F. Coltheart M. (2008). The role of signal detection and amplification in the induction of emotion by music. Behav. Brain Sci. 31, 597–598. 10.1017/S0140525X08005529

[B161] Trehub S. E. (2008). Music as a dishonest signal. Behav. Brain Sci. 31, 598–599. 10.1017/S0140525X08005530

[B162] Van-Goethem A. (2010). Affect regulation in everyday life: Strategies, tactics, and the role of music (Doctoral dissertation), Keele University.

[B163] Warrenburg L. A. (2020). Comparing musical and psychological emotion theories. Psychomusicology 30, 1–19. 10.1037/pmu0000247

[B164] Zeelenberg M. Pieters R. (2006). Social psychology and economics, in Manuscript Under Review, eds De Cremer D. Zeelenberg M. Murnighan K. (Mahwah, NJ: Lawrence Erlbaum Associates), 117–137.

[B165] Zentner M. Grandjean D. Scherer K. R. (2008). Emotions evoked by the sound of music: characterization, classification, and measurement. Emotion 8, 494–521. 10.1037/1528-3542.8.4.494 18729581

[B166] Zickfeld J. H. Schubert T. W. Seibt B. Blomster J. K. Arriaga P. Basabe N.. (2019). Kama muta: conceptualizing and measuring the experience often labelled being moved across 19 nations and 15 languages. Emotion 19, 402–424. 10.1037/emo0000450 29888936

