# Supplementary material for: The CODA Model: A Review and Skeptical Extension of the Constructionist Model of Emotional Episodes Induced by Music
Source: Front Psychol. 2022 Apr 13;13:822264. doi: 10.3389/fpsyg.2022.822264 (PMC9043863; doi:10.3389/fpsyg.2022.822264)
Supplement: Supplementary file 1 [file Data_Sheet_1.pdf]

frontiers

The CODA model: a review and skeptical extension of the constructionist model of emotional episodes induced by music

Thomas M. Lennie [1*], **Tuomas Eerola** [1]

[1] *Department of Music, Durham University, Durham, United Kingdom*

Correspondence*:
Thomas M. Lennie
thomas.m.lennie@durham.ac.uk

1 SUPPLEMENTARY MATERIAL

1.1 A. Parallel-competitive dual-process model

A third skeptical theory (Moors et al., 2017; Moors and Fischer, 2019) argues for the incorporation of dual-process models from cognitive and behavioural sciences into the emotion sciences, under the skeptical premise that the same mechanisms should be used to explain emotional episodes. This model is grounded in appraisal. However, like Russell (2003, 2012), it takes behaviour (emotional and non-emotional) as the "to be explained phenomena" through sub-components of "learning, motivation, action, and decision making" (Moors, 2017).

Dual-process theories typically propose two types of processes: stimulus-driven and goal-directed mechanisms. A goal-directed mechanism evaluates the functionality of multiple actions and initialises the action tendency with the greatest functionality in a given context. For example, an action tendency that may then become overt behaviour. Several cycles of multiple goals can run simultaneously to map changes in behaviour over time, as in cyclic cybernetic models of cognitive control (Inzlicht et al., 2015). Typically, dual-process theories adopt an 'interventionist' approach to the goal-directed mechanism, in which stimulus-driven processes are the default due to their simplicity and automatic nature. Goal-directed mechanism is more complex and less automatic but can intervene under certain conditions. The stimulus-driven mechanism has the risk of producing less optimal behaviour, while the goal-driven mechanism can correct for this to produce more optimal behaviour when the time and resources are available.

Moors et al. (2017) suggest an alternative "parallel-competitive" model in which they challenge the automatic / non-automatic distinction. They reason that the goal-directed mechanism can also be automatic[1]. They argue this through three core propositions. First, goal-directed operations can extend from simple (fewer possible action or outcome predictions) to complex (several action and outcome predictions). Second, goals can compensate for a lack in ideal operating conditions (e.g.,time or attention). Third, the goal-directed mechanism can recruit associative as well as rule-based operations. Empirical evidence for all three of these positions has emerged (Moors et al., 2017). The implication of this is that stimulus and goal-driven mechanisms will predominantly operate in parallel where the goal-directed mechanism should operate as the default cause of behaviour because the system will prioritise the process that leads to the most optimal predicted outcome. Moors et al. (2017) note how D-A theory is consistent with dual-process theories and attitude formation theories citing Smith and De Coster (2000) and Sloman (1996) respectively.

[1] They also acknowledge that under "poor operating conditions" the simplicity of the stimulus-driven mechanism can give it priority. However, this does not imply goal-directed mechanisms can not operate in these situations (Moors et al., 2017).

This model expands upon the typical appraisal processes though by placing within the goal-directed process an appraisal of 'utility'. This allows the most effective action (the action tendency with the best perceived outcome) to take priority over others, something that is not present in existing D-A theories. It makes no prediction about component interactions but acknowledges that both suggested skeptical options are possible.

In the broader concept of behavioural dual-process theories this model allows emotions to become a more central part of the broader decision making process, with the difference between colder and hotter cognitive processes being marked by the degree of goal-relevance a person places on a stimulus. Similar ideas have also been considered by others who incorporate animal as well as human behaviour (Eder and Hommel, 2013).

1.2 B. Constructionist model of musical emotions

Céspedes-Guevara's (2021) constructionist model of musical emotions proposes several mechanisms that reflect the influence upon arousal and valence (core-affect) independently. This is in conjunction with associative and appraisal mechanisms that influence the 'conceptual-act' (Barrett, 2006); the dynamic and fast integration of top-down knowledge with the sensory and affective information that defines core-affect. These two systems (core-affect & the conceptual-act) can be seen as separable by the aspects that inform them; (i) core-affect influenced by the acoustic cues within the music and (ii) the conceptual-act informed by the personal and social significance of an event.

Core-affect mechanisms influenced by acoustic cues inform arousal and valence separately – 'attunement' (p. 76). The concept of attunement is built around embodied cognition of music (internal mimicry of action), interaction with the affordances (Clarke, 2005) and states of action readiness (Frijda, 2009), and the dynamic neural resonance of musical sound (Flaig and Large, 2014). For arousal, four mechanisms are presented. This is multiple mechanisms (*neural resonance*, *bodily tuning*, *motor simulation*, and *prediction success*) viewed as equivalent phenomena at different neural timescales. The appraisal *prediction success* represents how successful the neural pattern predictions are at a cognitive level.

Valence is constructed around four mechanisms. Predominantly, intrinsic pleasantness (Scherer and Coutinho, 2013), and mediated by additional mechanisms of *mere exposure* (Huron, 2006), *evaluative conditioning* (Juslin, 2013), and *enculturation*. Céspedes notes that changes in valence are harder to predict from acoustic cues than arousal. Furthermore, valence emerges from the interaction between *intrinsic pleasantness* and the conceptual-act (*aesthetic preferences*, *goals*, *significance of events* etc.) possibly overriding such effects of 'primitive appraisals' (p. 83). However, little is mentioned as to how this interaction occurs.

The conceptual-act comprises two groups of mechanisms representing top-down information (*associative* and *rule-based appraisal* mechanisms), together allowing the categorisation of core-affect into a discrete musical emotion. These associative mechanisms include *visual imagery*, *episodic memories*, *semantic referents*, and *visual / verbal narratives*. More research into these mechanisms is required but Céspedes-Guevara suggests only episodic memory leads to induced emotion independently (2021). For other associative mechanisms it as necessary to invoke appraisal to provide situational relevance.

Three rule-based appraisal mechanisms are hypothesised by Céspedes-Guevara (*goal-relevance*, *control*, and *aesthetic judgment*) integrated from Scherer (2009) and Clore and Ortony (2000). *Goal-relevance* is described in relation to the situation, like most theories of musical emotions (Scherer and Coutinho, 2013; Juslin, 2019), where the emotion is not directly related to active music listening but to the task it facilitates

or impedes. However, he also notes that goals can be fulfilled by listening to music (e.g.,mood-regulation). This is seen as a conscious 'meta' decision where the abstraction of imagining how to achieve a goal is implied. *Control* is related to one's ability to stop or change the music, again related to situational evaluations. Both appraisals offer contextual assessment value. Yet, little discussion is given to broader conceptions of appraisal (e.g., Robinson, 2005; Silvia, 2005a).

The third appraisal is *aesthetic judgment*, consisting of an appraisal of the musician (e.g.,their skill level or creativity) and the music itself (e.g., its beauty, complexity, and challenge). It is suggested that aesthetic appraisals would differ culturally and that it could influence other capacities such as empathy. This *aesthetic judgment* mechanism is equivalent to the *norm-compatibility* appraisal (internal and cultural art norms, Scherer and Coutinho, 2013), with the additional appraisal of *agency* (music or performer). However, a list of aesthetic judgments can quickly form an ever-expanding list of evaluations (Egermann and Reuben, 2020) yet nearly all appraisal theories contend there should be a reasonably small number of appraisals. Notably, Céspedes-Guevara's concept of *aesthetic judgment* remains separate from the other appraisal mechanisms he proposes, both in being a meta-appraisal and leading to discrete aesthetic emotions. Moreover, no predictions about how perceptions of 'beauty' or 'challenge' may influence or interact with core-affect through a *conceptual-act* are offered. Aesthetic appraisal is not explicitly placed in a superior position to other mechanisms, as noted in BRECVEMA (2019), but does infer a special status as a conscious (meta) evaluation.

The final element of the model is 'attentional deployment.' This is divided into three levels: shallow, cognitively engaged but emotionally detached, and deep. This attentional process shapes the emotional experience of music and can be modified by choice. The first level (shallow) is situationally focused, has little personal significance, and is less likely to lead to an emotional reaction. In the second (un-emotional cognition) music is the active point of attentional focus but is not personally relevant. At the third (deep) level active meaning construction occurs through significant personal associative mechanisms and/or relevance to current goals. The music does not have to be actively attended to in the attentively deep level (but can be) and forms a meaningful part of a situation. All of these model elements lead to a complex process where musical cues induce changes in core-affect and are sculpted through the associative and/or rule-based appraisals to form discrete emotions under the right attentional attitude.

1.3 Constructionist model under a skeptical lens

Céspedes-Guevara's work has been instrumental in defining a constructionist model of musical emotions. Yet, there is still room for improvement and expansion based on our review of skeptical theories of emotion. His model incorporates some skeptical ideas (e.g., a move away from discrete emotions as purely stimulus driven) but still focuses upon the act of categorising. Céspedes-Guevara incorporates some appraisal mechanisms in his model as described above; several more than Barrett (2006) who contends 'appraisals are not literal cognitive mechanisms' (p. 40). He additionally notes the overlap between different mechanisms and appraisal mechanisms (Scherer and Coutinho, 2013) often defining them as equivalent at different neural timescales. Such an approach, which we endorse, allows the reader to link existing research on musical mechanisms with appraisal. Nonetheless, we seek to improve on this model with skeptical perspectives directed at 'folk' categories (**categorisation**), the construction and interaction of **components** and the narrow representation of **appraisal**.

1.3.1 Categorisation

Skeptical theories do not view emotion categories as meaningful scientific sets from which to group underlying cognitive mechanisms. Instead, they seek to remove the ever-expanding number of mediating mechanisms and focus upon the components of the emotion process to generate hypotheses shifting the focus to consciousness and behaviour. Céspedes-Guevara's continued focus on categorisation, despite being more diverse and granulated than basic emotions, defends distinctions between concepts like utilitarian and aesthetic emotions thus the need to invoke additional mechanisms such as aesthetic judgments. Emotions like *interest* and *surprise* are not considered utilitarian emotions in this construction because they are not clearly valenced. However, the literature on aesthetic emotions (Tan, 2000) marks them as highly important. This highlights the problem research has in undertaking the study of aesthetic emotions and further exemplifies why much research chooses to separate aesthetic experience from the rest of emotion research.

In acknowledging the skeptical aim to recenter research upon the components of an emotional episode, rather than the organisation of emotional space, the skeptical perspective offers a clear advantage in this respect. Research into aesthetic and utilitarian emotions can be neatly placed within the same mechanisms as general cognition. Goal-directed accounts may form a more valid scientific set in which to distinguish such concepts; that is embodied and enactive goal-directed affordances discussed in our CODA model.

1.3.2 Components

Skeptical theories further contend that the components of an emotional episode are more logically represented in multidimensional space. Within Céspedes-Guevara's model we can see such conceptions in core-affect, but not associative and appraisal mechanisms, which tend to be described in binary ways (the music produces a memory or it does not; the music is or is not goal-relevant). Adopting more nuanced dimensional accounts allows greater flexibility in the emotion process and additionally allows for more dynamic non-linear modelling, something Céspedes-Guevara himself notes as important.

The interaction between components is a point of contention in skeptical theories. Céspedes-Guevara makes little comment on component interactions apart from to note the complexity of multiple elements. The interaction between the conceptual-act and core-affect does not describe an actual interaction, just the process of labelling core-affect in relation to current situation. One exception to this is the valence dimension, where the conceptual-act mechanisms are noted as having a significant role in determining valence, in many cases overriding the initial mechanisms associated with the dimension (intrinsic pleasantness etc.). However, how this occurs, in what circumstances, or why certain appraisals should be superior to others is not described. We discuss below the situational and individual factors influencing stronger or weaker component interactions. Whether these component interactions form consistent patterns between events remains to be seen.

1.3.3 Appraisals

Skeptical theories all note the importance of appraisal, something we argue is missing from much of the music emotion literature. Céspedes-Guevara admirably has sought to rectify this and incorporated some rule-based appraisal mechanisms. However, exploration of the appraisal literature suggests not only can more be gleaned but it can lead to testable hypotheses applicable to several current music-emotion debates (discussed in our CODA model). Here, we offer a critique of current adaptions of appraisal.

First, it is not clear whether the *novelty* appraisal, a dimensional appraisal related to arousal, should be considered explicitly a novelty dimension or a novelty-familiarity continuum. The mediating mechanisms

Céspedes-Guevara links to valence are more closely tied to familiarity (enculturation / mere-exposure), so we assume these should be separate dimensions (see CODA model: novelty). We contend these appraisals should not be so explicitly divided between valence and arousal. A clear example comes from evidence of too much or too little novelty producing negative affect (Silvia, 2006). Similarly, for the *intrinsic pleasantness* appraisal associated with valence, there is evidence that extremely positive or negative ratings automatically increase arousal forming a V-shaped relationship (Kuppens et al., 2017). Together this suggests a more nuanced interaction between these dimensions and between appraisal and core-affect.

Second, we note the distinction between appraisals in core-affect and the conceptual-act. We find it strange that some appraisals (novelty & intrinsic pleasantness) should be reserved explicitly for acoustic cues in core-affect, while others (goal-relevance & control) for purely situational and personal aspects. We exclude *aesthetic judgment* from this distinction because it is representative of both though placed in conceptual mechanisms. Novelty and intrinsic pleasantness could very easily be applied to a situation as much research has shown (Scherer and Moors, 2019). Moreover, though less intuitively, we note that goal-relevance and control can equally be applied to acoustic features. Such examples come from Schiavio et al. (2017, goals) and Silvia (2005b, 2005a, 2006, control) and vocal acoustic cues (Nordström et al., 2017, goal-conduciveness, urgency, power, and norm-compatibility) discussed below. We simply note here that skeptical theories would argue such distinctions are not needed and appraisals are part of a dynamic interaction between individual and environment (sonic or situational).

Third, skeptical theories note that dimensional-appraisals can occur in both a rule-based and associative way[2]. Moreover, both processes are linked with multiple types of information processing. Céspedes-Guevara describes the appraisal mechanisms associated with the conceptual-act as explicitly rule-based, retaining the historic association between rule-based processing with conceptual and verbal-like information. Skeptical theories reject such dichotomies (Moors, 2017). Appraisals (including goals) can be seen as automatic, can be stimulus-driven in an embodied and enactive perceptual nature, and linked to multiple types of information (conceptual, propositional, perceptual, and sensory). Through this acknowledgment we remove constraints upon which types of processes appraisal can operate in. Such dichotomies between types of processes are closely tied to how familiar a stimulus is and form graded distinctions between ongoing rule-based and associative processing. For example, an unfamiliar tonal system is likely to be processed in a rule-based fashion, while a familiar rhythmic component may be processed associatively. We explore the distinctions between rule-based and associative process as graded distinctions further with examples in our CODA model.

Fourth, we identify the central role of *goals* in skeptical theories. Céspedes-Guevara acknowledges the many goals of musical engagement but refrains from allowing it a central role in core-affect or the acoustic environment. We note important evidence for links between arousal and *goal-relevance*, and *goal-conduciveness* with valence (Kuppens et al., 2012; Moors and De Houwer, 2001). Furthermore, we highlight that embodied and enactive goal-directed accounts of meaning construction can be acoustically relevant. The diverse and well documented reasons people have for engaging with music (Saarikallio, 2011; Randall and Rickard, 2017; Schäfer et al., 2013) can be constructed around the affordances of the acoustic environment (discussion and examples in CODA model). When such constructions are acknowledged, other mechanisms Céspedes-Guevara suggests as important mediators, such as *evaluative conditioning* (Juslin, 2013) in valence, may not be needed. As evidenced by Kolsseck, Yu, and Dickenson (2011) evaluative conditioning effects only occur under particular conditions, while a goal-directed interpretation

[2] Rule-based processes refer to the active processing of an appraisal or multiple appraisal outputs. *Associative* process refer to the learned association between a stimulus and appraisal outputs. Rule-based processes offer greater flexibility to the process while associative processes are more rigid.

remains more flexible (Moors et al., 2017; Moors, 2017). This removes the need for additional mediating mechanisms.

Finally, we note the contribution of appraisal to the overall system. We have cited the neurological evidence for more appraisals than are currently acknowledged (Brosch and Sander, 2013; Kafkas and Montaldi, 2014) and state that appraisals have been well acknowledged in cognitive and behavioural (animal & human) models (Eder and Hommel, 2013). Simply, by building on the skeptical notion that the same mechanisms should underpin both cognitive and emotional process (Dukes et al., 2021), we state that a greater incorporation of appraisal into music-emotion models it is a prerequisite for advancement in the field.

2 REFERENCES

Barrett, L. F. (2006). Solving the emotion paradox: Categorization and the experience of emotion. *Personality and social psychology review* 10, 20–46.

Brosch, T., and Sander, D. (2013). Comment: The appraising brain: Towards a neuro-cognitive model of appraisal processes in emotion. *Emotion Review* 5, 163–168.

Céspedes-Guevara, Julian. A. (2021). A constructionist theory of music induced emotions. *PsyArXiv*. Available at: psyarxiv.com/sfzm2.

Clarke, E. F. (2005). *Ways of listening: An ecological approach to the perception of musical meaning*. Oxford University Press, Oxford.

Clore, G. L., and Ortony, A. (2000). Cognition in emotion: Always, sometimes, or never. *Cognitive neuroscience of emotion*, 24–61.

Dukes, D., Abrams, K., Adolphs, R., Ahmed, M. E., Beatty, A., Berridge, K. C., Broomhall, S., Brosch, T., Campos, J. J., Clay, Z., et al. (2021). The rise of affectivism. *Nature Human Behaviour* 5, 816–820. doi:10.1038/s41562-021-01130-8.

Eder, A. B., and Hommel, B. (2013). Anticipatory control of approach and avoidance: An ideomotor approach. *Emotion Review* 5, 275–279.

Egermann, H., and Reuben, F. (2020). “Beauty is how you feel inside”: Aesthetic judgments are related to emotional responses to contemporary music. *Frontiers in Psychology* 11, 2959.

Flaig, N., and Large, E. W. (2014). Dynamic musical communication of core affect. *Frontiers in psychology* 5, 72.

Frijda, N. H. (2009). Emotion experience and its varieties. *Emotion Review* 1, 264–271.

Huron, D. (2006). *Sweet anticipation: Music and the psychology of expectation*. MIT press, London, UK.

Inzlicht, M., Bartholow, B. D., and Hirsh, J. B. (2015). Emotional foundations of cognitive control. *Trends in cognitive sciences* 19, 126–132.

Juslin, P. N. (2013). From everyday emotions to aesthetic emotions: Towards a unified theory of musical emotions. *Physics of life reviews* 10, 235–266.

Juslin, P. N. (2019). *Musical emotions explained: Unlocking the secrets of musical affect*. Oxford University Press.

Kafkas, A., and Montaldi, D. (2014). Two separate, but interacting, neural systems for familiarity and novelty detection: A dual-route mechanism. *Hippocampus* 24, 516–527.

Klossek, U. M., Yu, S., and Dickinson, A. (2011). Choice and goal-directed behavior in preschool children. *Learning & behavior* 39, 350–357.

Kuppens, P., Champagne, D., and Tuerlinckx, F. (2012). The dynamic interplay between appraisal and core affect in daily life. *Frontiers in psychology* 3, 380–388.

Kuppens, P., Tuerlinckx, F., Yik, M., Koval, P., Coosemans, J., Zeng, K. J., and Russell, J. A. (2017). The relation between valence and arousal in subjective experience varies with personality and culture. *Journal of personality* 85, 530–542.

Moors, A. (2017). Integration of two skeptical emotion theories: Dimensional appraisal theory and Russell's psychological construction theory. *Psychological Inquiry* 28, 1–19.

Moors, A., Boddez, Y., and De Houwer, J. (2017). The power of goal-directed processes in the causation of emotional and other actions. *Emotion Review* 9, 310–318.

Moors, A., and De Houwer, J. (2001). Automatic appraisal of motivational valence: Motivational affective priming and simon effects. *Cognition & Emotion* 15, 749–766.

Moors, A., and Fischer, M. (2019). Demystifying the role of emotion in behaviour: Toward a goal-directed account. *Cognition and Emotion* 33, 94–100.

Nordström, H., Laukka, P., Thingujam, N. S., Schubert, E., and Elfenbein, H. A. (2017). Emotion appraisal dimensions inferred from vocal expressions are consistent across cultures: A comparison between australia and india. *Royal Society open science* 4, 170912.

Randall, W. M., and Rickard, N. S. (2017). Reasons for personal music listening: A mobile experience sampling study of emotional outcomes. *Psychology of Music* 45, 479–495.

Robinson, J. (2005). *Deeper than reason: Emotion and its role in literature, music, and art*. Oxford University Press on Demand.

Russell, J. A. (2003). Core affect and the psychological construction of emotion. *Psychological review* 110, 145–172.

Russell, J. A. (2012). "From a psychological constructionist perspective," in *Categorical versus dimensional models of affect: A seminar on the theories of panksepp and russell*, eds. P. Zachar and R. D. Ellis (John Benjamins Publishing), 79–118.

Saarikallio, S. (2011). Music as emotional self-regulation throughout adulthood. *Psychology of music* 39, 307–327.

Schäfer, T., Sedlmeier, P., Städtler, C., and Huron, D. (2013). The psychological functions of music listening. *Frontiers in Psychology* 4, 511. doi:10.3389/fpsyg.2013.00511.

Scherer, K. R. (2009). The dynamic architecture of emotion: Evidence for the component process model. *Cognition and emotion* 23, 1307–1351.

Scherer, K. R., and Coutinho, E. (2013). "How music creates emotion: A multifactorial process approach," in *The emotional power of music: Multidisciplinary perspectives on musical arousal, expression, and social control*, eds. F. B. Cochrane T and K. R. Scherer (Oxford University Press Oxford, UK), 121–145.

Scherer, K. R., and Moors, A. (2019). The emotion process: Event appraisal and component differentiation. *Annual Review of Psychology* 70, 719–745.

Schiavio, A., Schyff, D. van der, Cespedes-Guevara, J., and Reybrouck, M. (2017). Enacting musical emotions. Sense-making, dynamic systems, and the embodied mind. *Phenomenology and the Cognitive Sciences* 16, 785–809.

Silvia, P. J. (2006). Artistic training and interest in visual art: Applying the appraisal model of aesthetic emotions. *Empirical studies of the arts* 24, 139–161.

Silvia, P. J. (2005a). Cognitive appraisals and interest in visual art: Exploring an appraisal theory of aesthetic emotions. *Empirical studies of the arts* 23, 119–133.

Silvia, P. J. (2005b). What is interesting? Exploring the appraisal structure of interest. *Emotion* 5, 89–102.

Sloman, S. A. (1996). The empirical case for two systems of reasoning. *Psychological bulletin* 119, 3–22.

Smith, E. R., and DeCoster, J. (2000). Dual-process models in social and cognitive psychology: Conceptual integration and links to underlying memory systems. *Personality and social psychology review* 4, 108–131.

Tan, E. S. (2000). "Emotion, art, and the humanities," in *Handbook of emotions*, eds. M. Lewis and J. M. Haviland-Jones (Guilford Press, New York), 116–134.
